# Quality Assessment of Burdekin Plum (*Pleiogynium timoriense*) during Ambient Storage

**DOI:** 10.3390/molecules28041608

**Published:** 2023-02-07

**Authors:** Gengning Chen, Michael E. Netzel, Sandra Milena Olarte Mantilla, Anh Dao Thi Phan, Gabriele Netzel, Dharini Sivakumar, Yasmina Sultanbawa

**Affiliations:** 1 ARC Industrial Transformation Training Centre for Uniquely Australian Foods, Queensland Alliance for Agriculture and Food Innovation, The University of Queensland, Brisbane, QLD 4068, Australia; 2 Department of Horticulture, Tshwane University of Technology, 0001 Pretoria West, South Africa

**Keywords:** fruits, *Pleiogynium timoriense*, storage, phenolic compounds, sensory

## Abstract

*Pleiogynium timoriense*, commonly known as Burdekin plum (BP), is among many Australian native plants traditionally used by Indigenous people. However, only limited information is available on the nutritional and sensory quality of BP grown in Australia as well as its changes during storage. Therefore, this study evaluated the quality of BP during one week of ambient storage (temperature 21 °C, humidity 69%). Proximate analysis revealed a relatively high dietary fiber content in BP (7–10 g/100 g FW). A significant reduction in fruit weight and firmness (15–30% and 60–90%, respectively) with distinguishable changes in flesh color (ΔE > 3) and an increase in total soluble solids (from 11 to 21 °Brix) could be observed during storage. The vitamin C and folate contents in BP ranged from 29 to 59 mg/100g FW and 0.3 to 5.9 μg/100g FW, respectively, after harvesting. A total phenolic content of up to 20 mg GAE/g FW and ferric reducing antioxidant power of up to 400 μmol Fe^2+^/g FW in BP indicate a strong antioxidant capacity. In total, 34 individual phenolic compounds were tentatively identified in BP including cyanidin 3-galactoside, ellagic acid and gallotannins as the main phenolics. Principle component analysis (PCA) of the quantified phenolics indicated that tree to tree variation had a bigger impact on the phenolic composition of BP than ambient storage. Sensory evaluation also revealed the diversity in aroma, appearance, texture, flavor and aftertaste of BP. The results of this study provide crucial information for consumers, growers and food processors.

## 1. Introduction

Currently, around 30 plant species account for more than 90% of plant-based food intake by humans [1]. Nonetheless, while there are over 6000 edible plants in Australia, only a few have been studied and commercialized [2]. In recent years, there has been an increasing awareness about the need to study and use underutilized plants which not only complement the modern diet but also help to preserve biodiversity [3]. As the global demand for natural and functional food products is growing steadily, there are many opportunities for utilising native Australian plants for a sustainable functional food market [2]. For example, the Kakadu plum (*Terminalia ferdinandiana*), high in vitamin C and ellagitannins, with pronounced antioxidant and antimicrobial properties, has been used in seafood preservation [4]. Carao (*Cassia grandis*), a plant native to central America, has been used for iron fortification in food products due to its high iron content [5]. Phytonutrients such as vitamins, minerals and phenolics are natural compounds found in plant foods [6]. Besides their functional application in food, phytonutrients, and especially phenolics in plants, can have a positive impact on human health [7]. Therefore, an increased intake of fresh fruits and vegetables helps to maintain health and can reduce the risk of chronic diseases [6]. However, levels of phytonutrients in modern human diets have decreased continuously, partly due to the reduction in the variety of foods derived from plant sources [6].

One of the lesser-known Australian native plants is *Pleiogynium timoriense.* It belongs to the Anacardiaceae family, which includes several economically important crops such as mango, cashew and pistachio [8]. This species is mainly distributed in coastal areas in Queensland (Australia) and also extends to neighbouring Indo-Pacific countries [9]. Only one species of *Pleiogynium* is recognized in Australia [9,10]; however, this species is phenotypically different in its fruit size, color and the hairiness of its leaves [10,11,12]. Other synonyms of *P. timoriense* are *P. cerasiferum* and *P. solandri* [13].

The fruits of Burdekin plum (BP) were traditionally consumed by Aboriginal Australians and were once popular among the public in north Queensland [14,15]. The fruits ripen between autumn and spring in Australia [15] and resemble flattened plums [16]. They are hard, sour and astringent when harvested. Traditionally, they are kept for a few days until the flesh turns slightly soft to obtain a more palatable taste [8,12,15]. Previous research showed the fruits are a valuable source of fiber, Ca, Zn and anthocyanins (bioactive polyphenols), and have a strong antioxidant capacity [17,18,19]. Recently, the phenolic composition of Burdekin plums cultivated in Egypt has been studied and 25 phenolic compounds, such as galloylquinic acid, gallic acid, ellagic acid, catechin and quercetin, were identified in the pericarp [20]. The fruit extract has also been found to have in vitro anti-inflammatory and anti-cancer effects [21]. 

However, the phenolic composition is known to vary depending on the growing location and environment [22]. In addition, fruits are metabolically active organisms and undergo physiological changes after harvesting, which leads to changes in the nutritional composition as well as physicochemical and organoleptic properties [23]. For example, an increase in total soluble solids was observed during the ambient storage of mangos [24]. Moreover, fruits usually experience a reduction in weight and firmness during storage [25], as well as an improved organoleptic quality, especially after short term storage [26]. To the best of our knowledge, there is no scientific information published on the changes in the quality of BP during the critical period between harvest and “having a more palatable taste”. Therefore, the present study aimed to evaluate BP harvested in Australia in terms of their physicochemical characteristics, phenolic composition and sensory properties, as well as the impact of ambient storage on these “parameters”.

## 2. Results and Discussion

### 2.1. Physicochemical Characteristics of Burdekin Plum

#### 2.1.1. Weight and Size

The BP fruits are oblate, resembling a flat prunus with a bigger equatorial diameter than vertical diameter. The sizes and weights of BP harvested from seven trees were summarized in Table 1. The size of the fruits varied with an equatorial diameter ranging from 32 to 42 mm and a vertical diameter ranging from 25 to 33 mm. The whole fruit weighed between 16 and 37 g with a flesh–stone ratio of 1.6 to 2.6. Fruit samples from Y2 had the highest flesh–stone ratio and smallest size while that from S1 had the lowest flesh–stone ratio and relatively big size. The dimensions of BP were within the ranges reported previously [12,15]. The flesh–stone ratio is much lower than that of commercial mangos [27] and plums [28], which could be a challenge for marketing in terms of flesh yield.

#### 2.1.2. Proximate Composition of Burdekin Plum

The proximate composition of the studied BP is shown in Table 2, which is similar to that reported previously [17]. The moisture content ranged from approximately 67 to 77 g/100 g FW and is slightly lower than that of cultivated fruits (usually around 85% or higher) [23]. However, this is a common feature of wild fruits [29]. Protein and fat were relatively low, ranging between 0.5–1.7 g/100 g FW and 0.6–1.8 g/100 g FW, respectively. Available carbohydrate (11–19 g/100 g FW) was the major macronutrient. The dietary fiber content ranged from approximately 7 to 10 g/100 g FW, which was similar to that reported by Said, et al. [30] while lower than the 18% reported by Brand-Miller, James and Maggiore [17]. However, it was considerably higher than that in cultivated plums, mangos and grapes (1–2%) [31], which is another characteristic of wild fruits [29]. Furthermore, BP can be considered an excellent source of fiber as one serving can provide more than 6 g dietary fiber [32]. Dietary fiber intake has been associated with many health benefits including enhancing satiation, regulating the gut microbiome and reducing the risk of cardiovascular diseases and diabetes [33].

#### 2.1.3. Changes in Weight, Firmness and Color during Storage of Burdekin Plum

Fruit weight, firmness and color changes during storage are shown in Table 3. All fruits stored at ambient conditions had a significant weight loss on day 7, with a loss between 15% and 30%. The weight loss of fruits harvested in Brisbane was lower than that in fruits from Cairns (weight loss in ascending order: S1 < S3 < S4 < S2 < Y3< Y1 < Y2). 

The fruit firmness was between 28 N and 42 N on day 0. By day 4, firmness was reduced significantly, especially in Y2 and S2 (below 10 N). The majority of fruits had a firmness below 10 N by day 7, except Y1, which had around 16 N. Overall, fruits lost over 60% to 90% of their original firmness by day 7, compared to day 0. Loss of firmness is a common phenomenon in fruits stored at ambient conditions, which is the result of the programmed ripening process [34]. This process is usually associated with the loss of moisture through transpiration, cell wall and starch degradation by enzymes and decreased cell turgor pressure [35,36]. 

**Figure 1:**
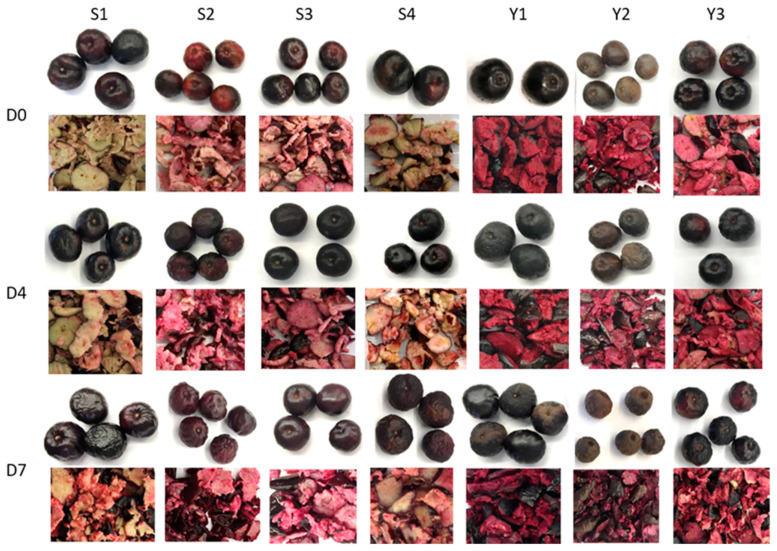
Whole fruits and flesh of the studied BP from seven trees (S1, S2, S3, S4, Y1, Y2, Y3) at three storage days (D0, D4, D7).

The peel color for most samples was dark maroon except for samples from Y1, Y2 and S2 (Figure 1). The peel of Y1 was black and distinguished from the rest by having the lowest redness compared to the rest of the samples. Y2 had a brown peel and was highest in lightness and yellowness. S2 had a lighter maroon peel and ranked highest in redness. The lightness of the peel was reduced by day 7, except for Y2. Generally during fruit storage, the lightness was reduced due to the loss of moisture and changes in the microstructure, leading to less light scattering [37]. The redness and yellowness were reduced on day 7 for S1, S2 and S3, while no significant changes could be observed in the other fruits. In terms of flesh color, all fruits experienced a reduction in lightness during storage, except S3, which had a non-significant (*p* > 0.05) reduction in flesh lightness on day 7. The changes in flesh redness varied, with S1 and S2 having an increased (*p* < 0.05) redness, while Y1, Y2 and Y3 having a reduced (*p* < 0.05) one. The yellowness was reduced (*p* < 0.05) in S1, S2, S3 and Y1 on day 7, while no significant (*p* > 0.05) changes occurred in the other samples. Furthermore, the total color difference ΔE of the peel and flesh color between different storage days was calculated [38]. Generally, very distinct differences can be observed when ΔE is larger than 3 [39]. Overall, the flesh color changes during storage were very distinct and perceivable (ΔE > 3), while this was less pronounced in the peel color. 

#### 2.1.4. Changes in TSS, pH and TA during Storage of Burdekin Plum

The changes in TSS, pH and TA are summarized in Table 4. TSS was similar on day 0 with an average of around 11 °Brix. However, TSS increased in all samples during storage reaching around 21 °Brix on day 7. The pH of the samples ranged from 2.8 to 3.8. No significant (*p* > 0.05) change in pH was observed during the ambient storage with the exception of S3, which increased (*p* < 0.05) on day 7 compared to day 0. TA in most samples was unaffected by the ambient storage ranging from 2.4 to 5.3% citric acid, except for S4 and Y1, which had a slight increase of TA on day 4. The sugar–acid ratio (TSS/TA) also increased on day 7. An increase in TSS in fruits is usually correlated to a better (sweeter) taste [40]. This is caused by soluble sugars generated by hydrolysis of polysaccharides including pectin and starch [41,42]. However, no further increase or decrease in TSS occurs as soon as the available polysaccharides are depleted, and the generated sugars are “consumed” for respiration and other metabolic activities [43].

Compared to European or Japanese plums, BP are more acidic and less sweet with higher TA [44]. However, when compared to the Davidson plum, another Australian native fruit, which is very acidic, BP has a higher pH and TSS and lower TA [45]. It should be noted that both stable acid content during storage and fluctuation in acidity during storage has been reported in the literature [43,46,47,48]. The concentration of organic acids is the result of organic acid synthesis, catabolism and tissue storage [49]. The increase in TA in some fruit samples may be caused by a ‘simple’ concentration effect due to the moisture loss of the samples during ambient storage [50], and/or a change in the respiratory metabolism, leading to an increased TA [51], and/or the inherent variation in the wild harvested fruits [52].

#### 2.1.5. Vitamin C and Folate of Burdekin Plum

Vitamin C is not only a powerful antioxidant, but also essential for many biochemical pathways and reactions in the human body, maintaining a healthy skin and preventing scurvy. Fruits and vegetables are the main dietary sources for vitamin C [53]. The changes of vitamin C in BP during ambient storage are shown in Table 5. On day 0, the vitamin C content in the fruits ranged from 29 to 59 mg/100 g FW, which was similar to that in mangos (13–93 mg/100 g FW) [54]. The S3 samples had the highest vitamin C content, whereas the Y1 the lowest. However, at the end of the 7-day storage, vitamin C content decreased to between 15 and 50 mg/100 g FW, which is still considered higher than that reported for Japanese plums (3–10 mg/100 g FW) [55]. Fruits can accumulate vitamin C while ripening, which can explain the slight increase of vitamin C in some samples on day 4 [56,57,58]. However, the loss of vitamin C on at the end of storage could be due to cell disruption and oxidation [59].

Folate is still a critical vitamin in many countries and a deficiency can cause neural development disorders and neurodegenerative diseases [60]. The folate content of the studied BP samples ranged from 0.3 to 5.9 μg/100 g FW. Compared to the recommended dietary intake (RDI) of folate for adults (400 μg/day in Australia) and other fruits and vegetables, such as mango (40 μg/100 g FW), strawberries (60–150 μg/100 g FW) and spinach (190 μg/100 g FW) [61], BP does not represent a relevant dietary source of folate. 5fTHF and 5mTHF were the predominant folate derivatives in the studied BP samples. 5mTHF is also the main folate derivative in many other fruits and vegetables [60,62]. Since the folate content in the studied BP samples was relatively low, no further analysis of the storage samples was carried out. 

#### 2.1.6. Identification of Phenolic Compounds

Phenolic compounds play a crucial role in the bioactivity and health benefits of plant food [63], including BP. Compounds were identified based on their *m*/*z*, characteristic MS2 fragments, mass spectra and retention time either compared to commercial standards or those reported in the literature [64]. Thirty-seven compounds were tentatively identified, with sixteen confirmed by commercial (reference) standards. The respective mass chromatograms and mass spectra are shown in Figures S1 and S2, respectively.

Seven anthocyanins were detected in positive ionization mode, as shown in Table 6. The peak at 6.84 min with a molecular ion of *m*/*z* 419 and MS2 fragment ion of *m*/*z* 287 by losing a pentosyl group (132Da) is characteristic of a cyanidin glycoside and was tentatively identified as cyanidin 3-O-arabinoside [64]. Two isomeric peonidin 3-hexoside were tentatively identified at 7.21 min and 7.46 min, as they had identical molecular ions of *m*/*z* 463 and produced MS2 fragment ions of *m*/*z* 301 by losing a hexosyl group (162 Da). A fragment ion of *m*/*z* 301 is characteristic of peonidin [64]. 

Twenty-seven non-anthocyanin phenolic compounds were detected in a negative ionization mode and summarized in Table 7. Phenolic acids were found in free form and esters with glycosides and quinic acids, especially gallic acid and gallotannins. Gallotannins are esters of one or more gallic acid molecules with a polyol such as glucose and quinic acid [65]. The compound that eluted at 1.32 min was tentatively identified as galloyl glucose. It had a molecular ion of *m*/*z* 331 and main MS2 fragments of *m*/*z* 313 after the loss of water (18 Da), *m*/*z* 271 and *m*/*z* 211 by losing one and two formaldehyde groups (CH_2_O) from the glucose molecule, and *m*/*z* 169 (deprotonated gallic acid) by losing a hexosyl group (162 Da) [66]. In addition, the characteristic fragment ion of gallic acid (*m*/*z* 125) was detected [67]. Three isomeric digalloyl glucose compounds were tentatively assigned to peaks at 5.4, 5.55, 5.8 min. They all had a parent ion of *m*/*z* 483 with secondary fragments of *m*/*z* 313 due to the loss of gallic acid (170 Da), *m*/*z* 271, *m*/*z* 211 and *m*/*z* 169, that were similar to the fragmentation pattern of galloyl glucose [67]. Similarly, six peaks at 6.03, 6.33, 6.52, 7.1, 7.5 and 7.7 min were tentatively assigned to trigalloyl glucose and its isomers. They all had a parent ion of *m*/*z* 635 and fragmentation patterns which were similar to digalloyl glucose [68,69]. Gallotannins including trigalloyl glucose and tetragalloyl glucose, which have been found in plants of the same family including mango and pistachio [70,71], were identified for the first time in BP. Two compounds eluted at 1.61 min and 1.88 min exhibited identical molecular ions of *m*/*z* 343 but had slightly different MS2 fragmentation patterns. One produced a MS2 fragment ion of *m*/*z* 191 by losing a galloyl moiety (152 Da) and a fragment ion of *m*/*z* 85, which was tentatively matched with the fragmentation pattern of 5-galloylquinic acid. The other produced MS2 fragment ions of *m*/*z* 169, *m*/*z* 173 and *m*/*z* 191, which resembles the fragmentation pattern of 4-galloylquinic acid [72].

Besides anthocyanins, flavanols and flavonols were the main flavonoids (tentatively) identified in the BP samples. The presence of catechin and epicatechin was confirmed using commercial standards. The peak at 8.51 min had a molecular ion of *m*/*z* 441 and MS2 fragments of *m*/*z* 169 and *m*/*z* 289, corresponding to deprotonated gallic acid and catechin or epicatechin, respectively. Further breakdown of these two fragments produced fragmentation ions of *m*/*z* 125 and *m*/*z* 245, due to the loss of one carbon dioxide (44Da) [73]. Based on this specific fragmentation pattern, the compound was tentatively identified as (epi)catechin gallate [74,75]. The compound eluted at 8.72 min had a similar fragmentation pattern as quercetin 3-glucoside and was tentatively identified as its isomer, possibly quercetin 7-glucoside or quercetin galactoside, which elutes earlier than quercetin 3-glucoside [76]. The peak at 8.83 min had a molecular ion of *m*/*z* 477 and the main fragment of *m*/*z* 301 by losing a glucuronic acid residue (176 Da) and was therefore tentatively identified as quercetin 3-O-glucuronide [76]. Quercetin 3-O-glucuronide was also found in mango and pistachio fruit and hull [70,77,78]. Similarly, two peaks at 9.49 and 9.65 min generated a molecular ion of *m*/*z* 461 and a secondary fragment of *m*/*z* 285 due to the loss of 176 Da which is characteristic of a glucuronic acid residue [79]. Based on the fragmentation pattern of the aglycon, the eluted compounds were tentatively identified as kaempferol glucuronide and luteolin glucuronide [76]. 

**Table 6:** High resolution accurate mass data of the identified anthocyanins in positive mode.

RT, min	Anthocyanins	Molecular Formula	Molecular Ion (*m*/*z*)	MS2 Fragments (*m*/*z*)			Reference
5.84	Delphinidin 3-galactoside	C_21_H_21_O_12_	465.1028	**303.0498**	304.0533	257.0446	standard
6.05	Delphinidin 3-glucoside	C_21_H_21_O_12_	465.1030	**303.0501**	304.0533	257.0438	standard
6.36	Cyanidin 3-galactoside	C_21_H_21_O_11_	449.1081	**287.0550**	288.058		standard
6.62	Cyanidin 3-glucoside	C_21_H_21_O_11_	449.1082	**287.0551**	288.0586		standard
6.84	Cyanidin 3-arabinoside	C_20_H_19_O_10_	419.0977	**287.0551**	288.0588		[64,80]
7.21	Peonidin 3-hexoside	C_22_H_23_O_11_	463.1241	**301.0707**	302.0739		[64]
7.46	Peonidin 3-hexosideisomer	C_22_H_23_O_11_	463.1241	**301.0707**	302.0739		[64]

RT: retention time, *m*/*z*: mass charge ratio, MS2 fragment: second stage mass spectrometry fragment. Quantification ion was highlighted in bold.

**Table 7:** High resolution accurate mass data of identified compounds in negative mode.

RT, min	Compounds	Molecular Formula	Molecular Ion (*m*/*z*)	MS2 Fragments (*m*/*z*)				Reference
0.92	Quinic acid	C_7_H_12_O_6_	**191.0559**	85.0294	93.0346	127.0401	137.0246	standard
0.95	Malic acid	C_4_H_6_O_5_	133.0142	**115.0036**	71.0138			standard
1.21	Citric acid	C_6_H_8_O_7_	191.0197	**111.0087**	191.0197	87.0087		standard
1.32	Galloyl glucose	C_13_H_16_O_10_	331.0670	169.0143	125.0244			[66]
1.50	Gallic acid	C_7_H_6_O_5_	169.0141	**125.0243**				standard
1.61	5-galloylquinic acid	C_14_H_16_O_10_	343.0668	**191.0560**	169.0143	85.0295		[72]
1.88	4-galloylquinic acid	C_14_H_16_O_10_	343.0667	**169.0142**	173.0455			
5.40	Digalloyl glucose	C_20_H_20_O_14_	**483.0774**	169.0142	125.0244	313.0563		[67,68,71]
5.55	Digalloyl glucose isomer I	C_20_H_20_O_14_	**483.0776**	169.0142	313.0563	125.0244		
5.80	Digalloyl glucose isomer II	C_20_H_20_O_14_	**483.0776**	169.0142	313.0563	125.0244		
5.56	Catechin	C_15_H_14_O_6_	**289.0716**	245.0818	109.0295	151.0401		standard
6.03	Trigalloyl glucose isomer I	C_27_H_24_O_18_	**635.0881**	169.0143	465.0675	313.0565	125.0244	[68,69]
6.33	Trigalloyl glucose isomer II	C_27_H_24_O_18_	**635.0881**	169.0143	465.0675	313.0565	125.0244	
6.52	Trigalloyl glucose isomer III	C_27_H_24_O_18_	**635.0884**	169.0143	465.0675	313.0565	125.0244	
6.87	Epicatechin	C_15_H_14_O_6_	**289.0716**	245.0823	109.0295	151.0401		standard
7.1	1,3,6-tri-o-galloyl-beta-D-glucose	C_27_H_24_O_18_	**635.0884**	169.0143	465.0675	313.0565	125.0244	standard
7.5	Trigalloyl glucose isomer IV	C_27_H_24_O_18_	**635.0884**	169.0143	465.0675	313.0565	125.0244	[69,71,81]
7.7	Trigalloyl glucose isomer V	C_27_H_24_O_18_	**635.0884**	169.0143	465.0675	313.0565	125.0244	
8.22	Tetragalloyl glucose	C_34_H_29_O_22_	**787.0993**	169.0143	635.0889	465.0671	125.0244	
8.24	Ellagic acid	C_14_H_6_O_8_	**300.9987**	229.0141	257.0092			standard
8.51	(Epi)catechin gallate	C_22_H_18_O_10_	441.0825	**169.0142**	289.0716	125.0244	245.0817	[74]
8.72	Quercetin 3-glucoside isomer	C_21_H_20_O_12_	463.0878	**300.0274**	271.0247	255.0298	151.0037	[76,77,82]
8.83	Quercetin glucuronide	C_21_H_18_O_13_	477.0673	**301.0352**	273.0407	151.0037		
8.85	Quercetin 3-glucoside	C_21_H_20_O_12_	463.0877	**300.0274**	271.0247	255.0299	151.0037	standard
9.49	Kaempferol glucuronide	C_21_H_18_O_12_	461.0723	285.0044	163.8401			[74,75]
9.65	Luteolin glucuronide	C_21_H_18_O_12_	461.0716	**285.0404**	175.0254			
9.85	Myricetin	C_15_H_10_O_8_	317.0299	**151.0036**	178.9985			standard
11.58	Quercetin	C_15_H_10_O_7_	**301.0350**	**151.0036**	178.9985	121.0294	273.0404	standard
11.67	Luteolin	C_15_H_10_O_6_	**285.0401**	**151.0036**	133.0294			standard
12.61	Kaempferol	C_15_H_10_O_6_	**285.0403**	**257.9139**	151.9234			standard

RT: retention time. Quantification ion was highlighted in bold.

#### 2.1.7. Quantification of Identified Compounds

The concentration of identified compounds is listed in Table S1. To identify the main contributors to the observed variation in BP samples of different storage days and trees, the quantified phenolic compounds were subjected to principal component analysis (PCA). The first three components explained 72.6% of the total variance in the dataset. 

PC1 described 30.6% of the observed total variance with positive loadings of the major anthocyanins and ellagic acid, and negative loading of digalloyl glucose, tetragalloyl glucose, trigalloyl glucose isomer IV&V, epicatechin gallate and catechin. PC2 accounted for 27.4% of the total variance and was characterized by positive loadings of quercetin 3-glucoside and its isomer, luteolin glucuronide and quercetin glucuronide, and negative loadings of 4-galloulquinic acid, trigalloyl glucose isomer I&II&III and epicatechin gallate. PC3 described 14.6% of the observed total variance and was associated with positive loadings of trigalloyl glucose and its isomer I&II&III, and negative loadings of 5-galloylquinic acid.

**Figure 2:**
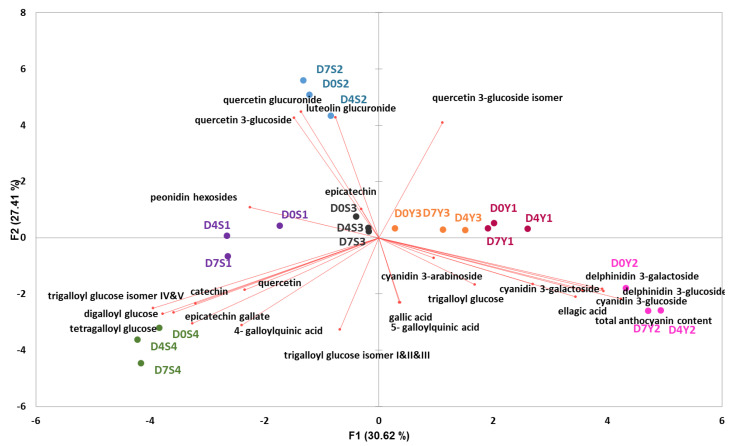

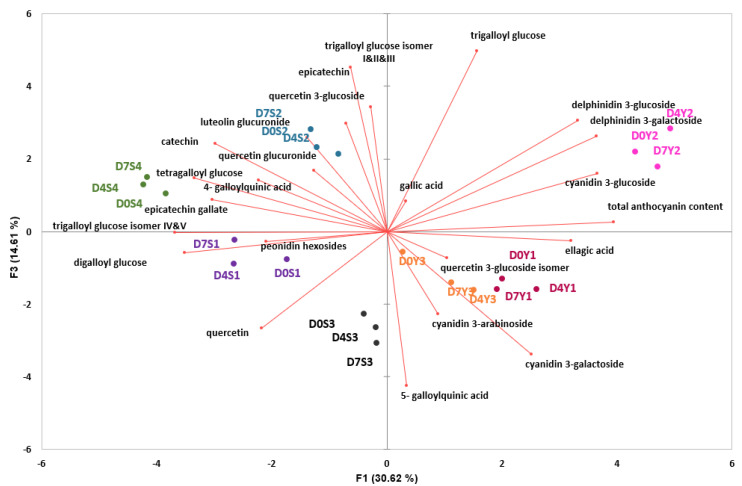
PCA bi− plots of phenolic compounds in Burdekin plums harvested from seven trees (S1, S2, S3, S4, Y1, Y2, Y3) on three storage days (D0, D4, D7).

The PCA plot (Figure 2) showed that fruits from seven trees could be separated by locating at different positions on the plot. Furthermore, the changes during storage were generally less pronounced compared to the variability among the trees. The Y2 samples scored highest in PC1, followed by Y1 and Y3, which were characterized by relatively high content in ellagic acid and major anthocyanins. The S2 samples scored high in PC2, which can be explained by their relatively high content in quercetin 3-glucoside, including its isomers, luteolin glucuronide and quercetin glucuronide. However, the S4 samples which were relatively high in digalloyl glucose, trigalloyl glucose isomer I & II & III & IV & V, tetragalloyl glucose, (epi)catechin gallate, catechin and 4-galloylquinic acid, scored lowest in PC1 and PC2. Furthermore, the S1 samples were located close to the negative side of PC1, whereas S3 samples were centered in the middle of the PC1 vs PC2 plot. The location of the S3 samples along the negative side of PC3 indicated a relatively low content of trigalloyl glucose, including its isomer I&II&III, and relatively high content of 5-galloylquinic acid.

The total anthocyanin content of the studied BP samples ranged from 240 to 2300 mg/kg FW. Cyanidin 3-galactoside was the dominant anthocyanin found in most samples, accounting for 80% (Y1) to 96% (S4) of total anthocyanins, except for Y2, which contained delphinidin 3-galactoside as the main anthocyanin (57% of total anthocyanins). Furthermore, cyanidin-3-arabinose and peonidin hexosides could be found in low concentrations in the studied BP samples (minor pigments). Y2, Y1 and Y3 had higher concentrations of cyanidin 3-glucoside, delphinidin 3-galactoside and delphinidin 3-glucoside than the other trees. Concentrations of cyanidin-3-arabinose and peonidin hexosides were similar among the BP samples and increased in most samples during the ambient storage (compared to day 0). However, an anthocyanin increase in all samples was observed on storage day 4. Overall, most ‘trees’ had an increase in total anthocyanins during 7 days of ambient storage, except Y1. Even so, the final anthocyanin concentration of the Y1 samples was just slightly below that of day 0, and still higher than the anthocyanin concentration in the samples harvested in Brisbane.

The S2 samples had a relatively low total anthocyanin content; however, the content was still comparable to or higher than that in other fruits such as plums (50–270 mg/kg FW) [83,84] and strawberries (200–500 mg/kg FW) [85]. The high anthocyanin content in the Y2 samples was comparable to that reported previously in this fruit and blueberries [19]. Blueberries are considered as a rich dietary source of anthocyanins, having an anthocyanin content from 660 to 3300 mg/kg FW [84]. The composition and content of anthocyanins are dependent on genotype and growth conditions [83,86], which can explain the variability in the anthocyanin composition and content in the studied BP samples. Cyanidin and delphinidin galactoside were the dominant anthocyanins in the studied BP samples, but also in bilberry (around 80%) [86]. Compared to other anthocyanins, these two anthocyanins have been found to possess a higher bioavailability [86]. Cyanidin 3-galactoside is also the main anthocyanin in pistachio hull (2.55 mg/100 g FW), accounting for 96% of total anthocyanins, and having a stronger antioxidant capacity than synthetic antioxidants such as BHT, BHA and Trolox [87]. The accumulation of anthocyanins during ambient storage and ripening is common in many fruits such as plums and berries [88], and usually leads to peel and flesh reddening [89]. The synthesis of anthocyanins during storage has also been found to be affected by the maturity of fruit when harvested [90]. Anthocyanin accumulation has been attributed to the upregulation of certain genes related to anthocyanin synthesis and transportation pathway [91]. However, the anthocyanin content is also affected by the downregulation of certain genes and degradation due to enzymatic or nonenzymatic factors, which can cause its reduction during storage [92,93].

The initial gallic acid content (2 to 50 mg/kg FW) was similar to that found in mango pulp (5 to 30 mg/kg FW) [94,95]. On storage day 7, all samples had a significant (*p* < 0.05) increase in gallic acid with concentrations of 7 to 210 mg/kg FW. The increase in gallic acid has also been observed in stored mango samples [96]. This can be explained by the release of gallic acid from hydrolysable gallotannins, which also explains the reduction in astringency, as gallotannins have stronger astringent taste than gallic acid [97]. Ellagic acid was within the range found in five mango cultivars (20 to 2000 mg/kg FW) [98]. Digalloyl glucose content ranged from 40 to 400 mg/kg FW, which was similar or higher than that reported in mango pulp (2–80 mg/kg DW) [71] and in Keitt mango peel (20 to 70 mg/kg FW) [99]. Trigalloyl glucose ranged from 20–200 mg/kg FW and was higher than that reported in Keitt mango peel (0.6–8 mg GAE/kg FW) [99]. Tetragalloyl glucose content ranged from 1.5 to 30 mg/kg and was similar to that in mango flesh (0.5–7 mg/kg DW) [71] and Keitt mango peel (around 10 mg/kg FW) [99]. Galloylquinic acid concentration ranged from 10 to 200 mg/kg FW and was similar to the levels in mango kernel [69] and Keitt mango peel which contains 5-galloylquinic acid between 20 to 30 mg/kg and total galloylquinic acid between 100 to 150 mg/kg [99]. Hydrolysable tannins, most likely gallotannins, constitute the major phenolics in mangos and have been found to exert various health benefits such as anti-inflammatory antidiabetic and antiviral effects [100,101,102]. Generally, gallotannins with a higher degree of galloylation (more than five) have higher antioxidant and antibacterial activities than gallotannins with a lower degree of galloylation [65]. The content of other identified flavonoids varied during the ambient storage but remained within a similar range. This ‘phenomenon’ has also been reported in other fruits during storage [103]. Catechin content ranged from around 2 to 40 mg/kg FW, a similar range to that reported in mango flesh (5–100 mg/kg FW) [71,104]. Epicatechin content ranged from 7 to 30 mg/kg FW, which was also comparable to that of mango flesh [105]. Quercetin ranged from 0.5 to 4 mg/kg FW, which was similar to that reported in mango puree [106]. Furthermore, the quercetin glucoside content was similar to that reported in mango pulp which is around 20 mg/kg [104,107].

The content of phenolic compounds in the BP samples collected from seven trees varied and the differences remained during the ambient storage. Furthermore, no consistent pattern could be observed in terms of the impact of storage on the phenolic compounds in the collected BP samples. However, similar results were reported in the literature and were attributed to the high fruit variations [98] and intraspecific variability [83,108,109]

#### 2.1.8. Antioxidant Capacity

The total phenolic content (TPC) of the BP samples during storage ranged from around 5 to 20 mg GAE/g FW (Table 8), which is comparable to that previously reported for this fruit [19,30] and considerably higher than that reported for mangoes (20–80 mg GAE/100 g FW) [107]. The ferric reducing antioxidant power (FRAP) values ranged from around 100 to 400 μmol Fe^2+^/g FW, which was also comparable to previously reported values of around 280 μmol Fe^2+^/g FW. In both assays, the S1 samples had the lowest values and the S4 samples the highest. Result showed that both TPC and FRAP were reduced on day 7, compared to day 0. The reduction in antioxidant activity has been observed in other fruits and is most likely caused by the senescence of the fruits during storage, which results in cell membrane disruption and accelerated oxidation of phenolic compounds by polyphenol oxidases [110]. Interestingly, a slight increase of the antioxidant capacity could be observed in some samples (e.g., S4 and Y3) on day 4. A similar ‘phenomenon’ has also been reported in strawberry, cherry and current. This has been attributed to the complex reactions taking place in fruits during postharvest storage leading to transient changes in phytochemical composition and increased antioxidant capacity [111].

### 2.2. Preliminary Sensory Evaluation

The descriptors from the sensory benchtop test summarized in Table 9 shows a wide diversity of sensory characteristics for the BP sampled, which is similar to the previous report about the big variations among BP [11]. Fruits varied in size, color, flavor and texture. Fruits from S1 and Y1 are relatively large compared to the rest. The peel color ranged from black, dark maroon to crimson, some glossy, some dull (Table 9). The flesh color ranged from beetroot red and pink to partly green. In terms of texture, some were juicy (Y2, S1, S3); some were dry and tough (Y1, Y3). The flavor of BP varies, resembling ripe plum, dried prunus, mulberry, blackberry, apples, kiwifruit and rose petals. The informal sensory evaluation of BP showed that most samples had a slightly fermented aroma, a dark maroon surface, a sour and fruity taste and a drying or astringent aftertaste, while distinct tree-to-tree variations existed. 

The slightly fermented aroma observed is common during fruit senescence and could be attribute to the ethanol formation, amino acid and fatty acid catabolism [48,112,113]. The sour taste can be explained by the relatively low pH and high TA compared to normal plums consumers used to consume. The drying and astringent taste and aftertaste, or even the bitter flavor in some samples, can be explained by the existence of acids and phenolics. Catechin and its derivatives, gallic acid and its derivatives, quinic acids and flavonols such as quercetin 3-glucoside have been reported to exhibit astringent sensation and bitter taste in grape, wine and tea [114,115], and were also found in BP. More rigorous descriptive profiling may be conducted in the future to assess the degree of difference among samples if necessary.

### 2.3. Limitations of the Present Study

Due to the complexities in accessing plants from Indigenous communities for scientific research [116], the research team was only able to secure fruits from seven trees from two different locations for this study. These two locations (or sites) were chosen after conversation and advice from our Indigenous project partners and the Sherwood Arboretum in Brisbane. Due to the small sample size and high tree to tree variation, the results were presented as per individual tree [17]. The statistical analysis was not as robust as originally anticipated due to the small sample size [117,118]. Despite these limitations, the present study provided novel and important data about Australian grown BP [19], including the intra-specific variation in fruit traits [119]. However, future studies should also focus on the genetic pool of BP, which can help us to better understand the observed variations and further elucidate its taxonomical classification [120,121].

## 3. Materials and Methods

### 3.1. Samples

Ripe fruits were harvested from seven trees, with three trees (Y1 to Y3) from Cairns (Queensland, Australia) and four trees (S1 to S4) from Brisbane (Queensland, Australia) in September 2021. Fruits from Cairns were air freighted to the laboratory under refrigeration within a day. Fruits from Brisbane were delivered immediately after harvest to the laboratory. Upon receiving, fruits were rinsed with tap water and cloth dried. At least 75 fruits from each tree with similar size and color and free from blemishes were selected for the storage trial and placed in trays on the shelves at ambient conditions (temperature 21.3 ± 0.8 °C, humidity 69.7 ± 6.9%). Fruits were sampled at 0, 4 and 7 days with 20 fruits from each tree at each time point for physicochemical analysis. The storage trial finished on day 7 when fruits turned soft on touch and some moldy fruits were observed. Sensory evaluation was conducted on 12 fruits from each tree after 7 days storage. An experimental design was shown in Figure S3.

### 3.2. Measurement of Physicochemical Properties

#### 3.2.1. Fruit Weight, Size and Color

Whole fruit weight and stone weight after removing flesh were measured using a laboratory scale (Sartorius CP224S, Goettingen, Germany) and the flesh–stone ratio was calculated. Fruit size, including vertical and equatorial diameter, was measured using a digital caliper (Craftright Engineering Works, Jiangsu, China). The vertical diameter was measured from the apical to the stem end of the fruit. The equatorial diameter was measured at the maximum width perpendicular to the vertical diameter. Peel and inner flesh color were measured using a Minolta CR-400 Chroma Meter (Konica Minolta, Osaka, Japan). Color space including a* (chromaticity coordinate from green to red), b* (chromaticity coordinate from blue to yellow) and L* (lightness) was recorded [122]. Total color difference ΔE between different storage days was calculated according to Pathare, Opara and Al-Said [38]

(1) $$\Delta E=\sqrt{\Delta {a^{*}}^{2}+\Delta {b^{*}}^{2}+\Delta {L^{*}}^{2}}$$

#### 3.2.2. Firmness

Firmness was measured using a texture analyzer (Ametek TA1, Largo, FL, USA) equipped with a 4 mm diameter cylindrical probe. Firmness was measured at the apical end of fruits at a loading speed of 0.5 mm/s to a depth of 2 mm and the maximum force (N) was recorded as firmness [123].

#### 3.2.3. Total Soluble Solids, pH and Titratable Acidity 

The milled fruit puree was used to measure total soluble solids (TSS), pH and titratable acidity (TA). TSS was measured using a digital refractometer (Atago, Tokyo, Japan), whereas pH and TA were measured using an automatic titration system (Metrohm 765 Karl Fischer Titrator system, Metrohm, Herisau, Switzerland). Titratable acidity was determined by titration with 0.1 N NaOH up to pH 8.2. TA was expressed as grams of citric acid equivalents per 100 g of fresh weight [124]. 

#### 3.2.4. Proximate Analysis

Fruit flesh with peel was analyzed for moisture by AOAC method 925.10. Fruit flesh with peel was freeze dried for 96 h using a freeze dryer (ScanVac CoolSafe 55–80 Superior, Vassingerød, Denmark). The freeze-dried fruit powder was stored at –20 °C for further analysis. Freeze dried fruit powder was analyzed for fat by method 960.39, protein by method 990.03, crude ash by method 923.03 and dietary fiber by method 985.29. The available carbohydrate content was calculated by subtracting moisture, protein, fat, ash and dietary fiber content from 100% according to AOAC [124]. 

#### 3.2.5. Vitamin C and Folate

Freeze dried fruit powder was analyzed for vitamin C using a Thermo Vanquish UHPLC-PDA system (Thermo Fisher Scientific, Waltham, MA, USA) with a Waters^®^ Acquity HSS T3 column (150 × 2.1 mm, 1.8 μm) (Waters, Rydalmere, NSW, Australia) according to the method reported by Phan et al. [125], with slight modifications. Briefly, 100 mg fruit powder was extracted with 2 mL solvent containing 8% acetic acid, 3% metaphosphoric acid and 1 mM ethylenediaminetetraacetic acid tetrasodium salt three times under vortexing, sonication, shaking and centrifugation. Supernatants were combined and filtered using a 0.22 um GHP membrane filter (Pall, Melbourne, VIC, Australia) before analysis. Dehydroascorbic acid (DHAA) in the sample was reduced using DL-Dithiothreitol. Total vitamin C (L-AA + DHAA) was determined at 245 nm and 25 °C with isocratic elution (aqueous 0.1% formic acid at 0.2 mL/min). 

Folate vitamers were analyzed following a stable isotope dilution assay according to the method described by Striegel, Chebib, Netzel and Rychlik [61], using a UHPLC-MS/MS (Shimadzu, Rydalmere, NSW, Australia), equipped with a Raptor ARC-18 column (Restek, Bellefonte, PA, USA). The folate derivatives measured included pteroylmonoglutamic acid (PteGlu), 5-methyltetrahydrofolate (5mTHF), 5-formyltetrahydrofolate (5fTHF), 10-formyl-pteroylglutamic acid (10f PteGlu) and tetrahydrofolate (THF).

### 3.3. Analysis of Phenolic Compounds

#### 3.3.1. Extraction

The freeze-dried fruit powders were extracted following the method described by Hong et al. [126]. In brief, freeze dried BP powder (0.2 g) was vortexed with 3 mL of 80% aqueous methanol containing 1% HCl. After centrifugation at 4000 rpm for 10 min at 4 °C (Eppendorf Centrifuge 5804, Eppendorf, Hamburg, Germany) the supernatant was collected and the residue was re-extracted two more times with 3 mL solvent each time, followed by a series of vortexing, sonication and shaking for 10 min at 200 rpm on a reciprocating shaker (RP1812, Paton Scientific, Victor Harbor, SA, Australia). After centrifugation, all supernatants were combined and stored at −35 °C until analysis. Extracts were filtered using a 0.22 um GHP membrane filter (Pall, Melbourne, VIC, Australia) before analysis.

#### 3.3.2. Ultra-High-Performance Liquid-Chromatograph and High Resolution/Accurate Mass Spectrometry (UHPLC-HRAM-MS/MS)

Identification and quantification of the compounds was performed using a Thermo Orbitrap Exploris™ 120 mass spectrometer equipped with a Thermo Vanquish™ UHPLC system (Thermo Fisher Scientific, Waltham, MA, USA). The chromatographic separation was based on the method by Hong, Phan and O’Hare [126], with slight modifications. Briefly, a Waters BEH C18 analytical column (150 × 2.1 mm, 1.8 μm particle size) (Waters, Rydalmere, NSW, Australia) was used with the system maintained at 50 °C for compound separation. The injection volume was 2 μL. The mobile phases consisted of A (LCMS grade water, acetonitrile and formic acid, 96:3:1, *v/v/v*) and B (acetonitrile and formic acid, 99:1, *v/v*) at a flow rate of 0.4 mL/min. The following gradient was used for B: 0% for 2 min, increased to 25% over 10 min, to 60% over 2.5 min, to 90% in 1.5 min and then held at 90% for 1 min, followed by recondition for 4.5 min before the next injection. A full MS scan (90–1000 *m*/*z*) was operated in positive and negative electron spray ionization (ESI) mode with a resolving power of 60,000 full widths at half maximum, followed by TOP-4 data dependent MS2 acquisition (first mass scan from 40, resolution of 15,000 FWHM at *m*/*z* 200 at the resolution of 15,000 with stepped collision energy at 15, 30 and 45 eV). Identification of phenolic compound was performed by matching their retention times and mass spectral data with those of the standard compounds and those reported in literature. For the compounds of interest, a product ion scan with an inclusion list of compounds (Table 6 in positive mode and Table 7 in negative mode) was conducted at a resolving power of 30,000 FWHM, mass tolerance of 5 ppm, and the stepped collision energy 15, 30, 45 eV. Quantification of the targeted compounds was based on the external calibration curves. External standards used included delphinidin 3-galactoside, delphinidin 3-glucoside, cyanidin 3-galactoside and cyanidin 3-glucoside, catechin, epicatechin, gallic acid, ellagic acid, quinic acid, citric acid, malic acid, quercetin, quercetin 3-glucoside, luteolin, kaempferol, myricetin and 1,3,6-tri-o-galloyl-beta-D-glucose (Merck/Sigma-Aldrich, Castle Hill, NSW, Australia). Software Thermo Xcalibur 4.0 and Tracefinder 5.1 (Thermo Fischer Scientific) were used for data acquisition and processing, respectively. Results were expressed as mg per kg of fresh sample weight (mg/kg FW). 

#### 3.3.3. Antioxidant Capacity

The total phenolic content (TPC) was determined using the Folin–Ciocalteu assay (Fredericks et al., 2013). In brief, the extracts were diluted appropriately with Milli-Q water. Blank (Milli-Q water) standard or sample was added into a 96-well plate (25 μL), followed by 125 μL 10% Folin–Ciocalteu reagent and 125 μL 7.5% sodium carbonate solution. The plate was incubated for 15 min and read at 750 nm using a spectrophotometer (Varioskan LUX Multimode Reader, Thermo Fisher Scientific Australia Pty Ltd., Scoresby, VIC, Australia). TPC was expressed as mg gallic acid equivalent (GAE) per g fresh weight (FW).

The ferric reducing antioxidant power (FRAP) assay was executed according to Netzel et al. [127], with slight modification. In brief, 30 μL of Milli-Q water and 20 μL the extract at appropriate dilution with Milli-Q water were mixed with 200 μL of FRAP reagent consisting of 2,4,6-tripyridyl-s-triazine (TPTZ) and ferric chloride in a 96-well plate. The plate was incubated for 8 min and the absorbance was measured at 593 nm. Results were expressed as μmol Fe^2+^ per g FW.

### 3.4. Preliminary Sensory Evaluation

A benchtop test sensory experiment was conducted to develop preliminary sensory descriptors of BP from seven trees at the end of the storage (fruits were stored for 8 days at ambient conditions), following the method by Shelat et al. [128], with slight modification. Briefly, informed consent from panellists was obtained before the evaluation. Twelve trained panellists with an average age of 48 years participated in one 90 min session for evaluation. Each panellist was presented with seven samples on individual small plates coded with a three-digit random number. Evaluation consists of providing descriptors for appearance, aroma, texture, flavor and aftertaste. Samples were evaluated at room temperature. Water and crackers were used as palate cleansers. Panellists were provided with the initial lexicon and instructed to eat fruits as they would normally do, being mindful of the big stone in the fruits. After tasting was completed, a discussion with the panel was led by the panel leader to generate descriptors by consensus for the seven samples.

### 3.5. Statistical Analysis

Data were processed using Microsoft Excel (Microsoft corporation, Washington, DC, USA) and XLSTAT 2022 (Addinsoft, Paris, France). Analysis of variance (ANOVA) followed by a Tukey’s multiple comparison test were used to compare differences between samples. A *p*-value < 0.05 was considered as statistically significant. PCA analysis was carried out for quantified phenolic compounds with results standardized based on Pearson’s correlation matrix.

## 4. Conclusions

Overall, the high dietary fiber content and the broad spectrum of phenolic compounds (mainly anthocyanins, ellagic acid and gallotannins) in BP are strong indicators of its potential health benefits and opportunities to market access. PCA indicated that tree to tree variation had a bigger impact on the phenolic compounds in BP than ambient storage. Ambient storage within one week is sufficient for Burdekin plums to turn soft for direct consumption. However, due to the relatively low flesh–stone ratio, further processing (such as turning into a powdered form) will be desirable to improve the marketability of Burdekin plums. These findings are important in terms of selecting fruits with specific traits for processing and/or product development, but also for consumption by consumers. Furthermore, Indigenous communities can use the results for tree selection and propagation.

## Figures and Tables

**Table 1:** Size and weight of the studied BP.

Samples	Equatorial Diameter (mm)	Vertical Diameter (mm)	Whole Fruit Weight (g)	Stone Weight (g)	Flesh Weight (g)	Flesh–Stone Ratio
Y1	42.3 ± 1.56 a	32.8 ± 0.81 a	36.6 ± 3.03 a	11.5 ± 1.11 b	25.1 ± 2.09 a	2.2 ± 0.14 bc
Y2	32.5 ± 1.25 e	25.9 ± 0.87 c	16.5 ± 1.28 d	4.7 ± 0.54 e	11.8 ± 1.01 e	2.6 ± 0.29 a
Y3	36.8 ± 1.30 c	29.1 ± 1.34 b	24.5 ± 2.53 c	7.0 ± 0.76 d	17.6 ± 1.98 c	2.5 ± 0.24 a
S1	42.2 ± 1.81 a	31.7 ± 1.03 a	34.8 ± 2.93 a	13.6 ± 0.77 a	21.3 ± 3.07 b	1.6 ± 0.27 e
S2	34.8 ± 1.38 d	28.2 ± 0.90 b	22.6 ± 4.00 c	8.0 ± 1.91 cd	14.6 ± 2.26 d	1.9 ± 0.32 de
S3	36.9 ± 0.57 c	25.7 ± 0.73 c	21.8 ± 0.98 c	7.4 ± 0.46 d	14.4 ± 0.77 d	1.9 ± 0.14 cd
S4	39.8 ± 1.60 b	29.3 ± 1.07 b	29.5 ± 2.38 b	8.9 ± 0.75 c	20.6 ± 1.91 b	2.3 ± 0.19 ab

Y1, Y2, Y3, S1, S2, S3, S4: BP fruits sampled from seven trees. Data are mean ± standard deviation (SD) (n = 10). Data without a common letter in each column indicate significant (*p* < 0.05) differences between samples using the Tukey (HSD) test.

**Table 2:** Proximate composition of the studied BP (g/100 g FW).

Sample	Moisture	Protein	Fat	Ash	Fiber	Carbohydrate
S1	69.3	0.9	0.7	1.2	8.4	19.5
S2	73.8	0.8	1.2	1.5	8.9	13.7
S3	72.5	0.7	1.2	1.0	7.1	17.5
S4	71.6	0.6	1.8	1.2	8.2	16.6
Y1	69.6	1.7	0.6	1.3	10.1	16.7
Y2	75.8	1.5	1.1	1.3	8.7	11.6
Y3	72.2	0.5	1.2	1.2	9.8	15.1

Data are mean (n = 2).

**Table 3:** Weight, firmness and color of the studied BP during ambient storage.

Test	Day	S1	S2	S3	S4	Y1	Y2	Y3
Firmness (N)	0	38.3 ± 3.31 ab	35.9 ± 5.15 abc	34.8 ± 3.06 bcd	32.1 ± 4.53 cd	42.0 ± 8.04 a	28.9 ± 4.55 de	28.5 ± 4.36 de
	4	30.6 ± 3.43 cd	9.1 ± 2.04 h	22.1 ± 6.80 f	23.5 ± 5.65 ef	33.4 ± 4.66 bcd	5.4 ± 1.21 h	16.5 ± 3.46 g
	7	4.4 ± 1.90 h	5.7 ± 1.47 h	5.6 ± 0.85 h	3.9 ± 1.07 h	16.8 ± 5.10 g	4.0 ± 1.18 h	9.4 ± 1.85 h
Weight (g)	0	32.8 ± 1.69 b	23.0 ± 1.77 fgh	22.5 ± 2.03 fghi	29.1 ± 1.64 bcd	38.5 ± 3.38 a	16.1 ± 1.51 kl	25.8 ± 2.06 def
	4	29.8 ± 2.07 bcd	19.6 ± 1.76 ghijk	19.8 ± 1.78 ghijk	25.6 ± 1.72 def	31.0 ± 3.17 bc	12.8 ± 1.28 lm	21.0 ± 2.08 ghij
	7	27.9 ± 1.79 cde	18.1 ± 1.91 jk	18.5 ± 1.71 ijk	23.8 ± 1.75 efg	27.8 ± 3.07 cde	11.3 ± 1.13 m	18.7 ± 2.15 hijk
Peel L*	0	26.6 ± 0.79 bc	28.2 ± 0.61 b	26.5 ± 0.60 bc	25.9 ± 0.57 cde	26.0 ± 0.56 cd	32.9 ± 1.13 a	26.0 ± 0.63 cd
	4	25.9 ± 0.72 cde	26.1 ± 0.28 cd	25.8 ± 0.42 cde	25.0 ± 0.70 cde	25.4 ± 0.42 cde	32.7 ± 1.84 a	25.5 ± 0.62 cde
	7	25.4 ± 0.48 cde	25.3 ± 0.77 cde	25.7 ± 0.40 cde	24.5 ± 0.48 de	24.1 ± 2.11 e	32.3 ± 1.57 a	24.7 ± 0.68 cde
Peel a*	0	6.4 ± 2.12 bcd	10.3 ± 2.32 a	6.5 ± 2.22 bc	4.8 ± 1.59 cdefg	2.1 ± 0.59 ghi	4.5 ± 0.61 cdefgh	2.3 ± 1.12 fghi
	4	5.1 ± 1.53 cdef	8.3 ± 2.21 ab	5.0 ± 0.57 cdef	3.4 ± 1.40 efghi	1.7 ± 0.29 hi	4.4 ± 1.02 cdefghi	2.5 ± 1.11 fghi
	7	3.8 ± 0.40 cdefghi	6.0 ± 1.59 bcde	3.6 ± 1.08 defghi	3.3 ± 1.03 efghi	1.6 ± 0.62 i	4.7 ± 0.91 cdefg	2.2 ± 1.02 fghi
Peel b*	0	3.0 ± 0.88 def	5.72 ± 1.26 bc	2.7 ± 0.65 ef	2.3 ± 0.69 ef	1.5 ± 0.16 f	7.2 ± 1.13 ab	1.4 ± 0.31 f
	4	2.5 ± 0.40 ef	4.67 ± 0.9 cd	2.2 ± 0.09 ef	2.2 ± 0.64 ef	1.6 ± 0.16 f	7.8 ± 1.84 a	1.7 ± 0.41 f
	7	2.1 ± 0.20 ef	3.53 ± 0.77 de	1.9 ± 0.22 ef	2.0 ± 0.31 ef	1.9 ± 0.82 ef	8.2 ± 1.80 a	1.8 ± 0.57 ef
Peel ΔE	0–4	1.5	3.1	1.8	1.7	0.8	0.7	0.6
	4–7	1.4	2.8	1.4	0.6	1.4	0.6	0.87
	0–7	2.9	5.7	3.1	2.1	2.1	1.2	1.4
Flesh L*	0	72.7 ± 0.83 a	59.1 ± 2.46 bc	61.5 ± 5.98 b	60.6 ± 0.99 b	30.2 ± 1.20 hi	26.9 ± 1.39 i	47.3 ± 3.68 efg
	4	65.8 ± 2.49 ab	47.0 ± 3.19 fg	56.6 ± 5.70 bcde	57.1 ± 3.61 bcd	29.5 ± 1.59 i	25.6 ± 0.58 i	45.1 ± 1.25 fg
	7	60.0 ± 4.26 b	46.3 ± 4.45 fg	50.3 ± 3.17 cdef	49.3 ± 2.51 def	24.4 ± 0.95 i	21.6 ± 1.99 i	39.6 ± 2.07 gh
Flesh a*	0	−1.7 ± 1.41 j	16.7 ± 0.49 fghi	22.2 ± 7.01 defgh	19.4 ± 1.14 defgh	37.2 ± 1.60 ab	30.6 ± 0.88 abcd	39.8 ± 2.95 a
	4	11.7 ± 2.85 hi	24.7 ± 6.08 cdef	21.5 ± 6.72 defgh	18.6 ± 6.51 efgh	33.7 ± 1.43 abc	28.0 ± 0.42 bcde	36.3 ± 4.85 ab
	7	6.3 ± 3.97 ij	27.3 ± 2.13 bcdef	12.9 ± 1.87 ghi	18.2 ± 1.77 efgh	23.9 ± 1.27 cdefg	23.0 ± 4.29 cdefg	29.1 ± 1.99 abcde
Flesh b*	0	23.9 ± 0.69 a	11.3 ± 1.39 efg	10.1 ± 1.25 efgh	16.8 ± 0.75 bcd	11.2 ± 1.25 efg	5.5 ± 1.70 hi	9.1 ± 1.37 efghi
	4	18.7 ± 2.11 bc	5.0 ± 2.3 hi	8.1 ± 2.52 fghi	17.1 ± 1.96 bcd	9.8 ± 2.01 efgh	4.6 ± 0.58 i	12.7 ± 1.37 def
	7	20.4 ± 2.64 ab	7.2 ± 2.23 ghi	5.6 ± 1.26 hi	14.2 ± 0.54 cde	6.7 ± 1.26 ghi	5.6 ± 1.02 hi	12.7 ± 2.11 def
Flesh ΔE	0–4	16.0	15.8	5.4	3.6	3.9	3.0	5.5
	4–7	8.1	3.5	11.0	8.4	11.5	6.5	9.1
	0–7	15.4	17.1	15.3	11.6	15.2	9.2	13.7

L*: lightness, a*: redness, b*: yellow. ΔE: total color difference. Data are mean ± SD (n = 5). Data without a common letter in each test indicate significant (*p* < 0.05) differences between samples using the Tukey (HSD) test.

**Table 4:** pH, TA and TSS of the studied BP during ambient storage.

Test	Day	S1	S2	S3	S4	Y1	Y2	Y3
pH	0	3.1 ± 0.17 defgh	3.3 ± 0.09 abcde	3.0 ± 0.10 efgh	2.8 ± 0.02 h	3.4 ± 0.13 abc	3.6 ± 0.17 a	2.9 ± 0.03 fgh
	4	3.0 ± 0.07 efgh	3.1 ± 0.09 defgh	2.9 ± 0.04 gh	2.8 ± 0.05 gh	3.2 ± 0.04 bcdef	3.4 ± 0.06 abc	2.8 ± 0.04 gh
	7	3.0 ± 0.09 defgh	3.3 ± 0.20 abcde	3.4 ± 0.06 abc	2.9 ± 0.08 gh	3.3 ± 0.08 abcd	3.4 ± 0.05 ab	3.1 ± 0.02 cdefg
TSS	0	10.7 ± 0.13 g	11.2 ± 0.31 fg	10.7 ± 1.03 g	12.4 ± 1.10 efg	11.5 ± 0.70 fg	11.5 ± 0.65 fg	12.5 ± 0.90 efg
(°Brix)	4	12.9 ± 0.88 efg	16.8 ± 1.14 cde	18.0 ± 0.47 bcd	19.3 ± 1.11 bcd	19.4 ± 1.24 bcd	17.2 ± 0.58 cde	15.8 ± 3.29 def
	7	18.5 ± 2.06 bcd	22.2 ± 2.81 ab	22.1 ± 3.42 ab	25.3 ± 0.70 a	19.8 ± 2.40 bcd	21.5 ± 0.70 abc	18.7 ± 1.10 bcd
TA	0	3.6 ± 0.12 defgh	4.2 ± 0.14 bcdef	3.5 ± 0.29 fgh	4.2 ± 0.30 bcdef	3.0 ± 0.28 gh	2.9 ± 0.59 h	4.6 ± 0.22 ab
(% citric acid)	4	3.7 ± 0.18 cdefgh	4.2 ± 0.13 bcdef	3.7 ± 0.35 defgh	5.1 ± 0.11 a	4.4 ± 0.19 abcde	3.6 ± 0.12 efgh	5.2 ± 0.12 a
	7	3.6 ± 0.18 efgh	4.5 ± 0.38 abcd	3.3 ± 0.20 gh	4.5 ± 0.49 abc	3.8 ± 0.20 bcdefg	3.5 ± 0.41 fgh	4.4 ± 0.28 abcde
TSS/TA	0	3.0 ± 0.10 fg	2.7 ± 0.03 g	3.1 ± 0.06 fg	3.0 ± 0.18 fg	3.8 ± 0.19 defg	4.0 ± 0.60 defg	2.7 ± 0.11 g
	4	3.5 ± 0.39 efg	4.0 ± 0.21 defg	5.0 ± 0.36 bcde	3.8 ± 0.15 defg	4.4 ± 0.31 cdef	4.8 ± 0.06 bcde	3.1 ± 0.63 fg
	7	5.2 ± 0.60 bcd	4.9 ± 0.27 bcde	6.7 ± 0.94 a	5.6 ± 0.71 abc	5.2 ± 0.91 abcd	6.2 ± 0.85 ab	4.3 ± 0.53 cdef

TA: titratable acidity, TSS: total soluble solids. Data are mean ± SD (n = 3). Data without a common letter in each test indicate significant (*p* < 0.05) differences between samples using the Tukey (HSD) test.

**Table 5:** Vitamin C and folate in the studied BP.

Vitamin	Day	S1	S2	S3	S4	Y1	Y2	Y3
Total Vitamin C (mg/100 g FW)	0	49.2 ± 0.85 cd	40.4 ± 0.10 efg	58.6 ± 1.03 b	56.7 ± 5.12 bc	29.2 ± 0.21 ijk	31.6 ± 0.82 hij	31.8 ± 0.29 hij
	4	49.3 ± 0.91 cd	41.5 ± 0.67 def	66.8 ± 5.40 a	43.7 ± 4.85 de	34.0 ± 2.90 fghij	35.0 ± 0.45 fghij	34.9 ± 3.01 fghij
	7	39.0 ± 0.96 efgh	33.1 ± 1.66 ghij	49.4 ± 3.84 cd	36.5 ± 0.76 efghi	21.1 ± 1.30 kl	27.2 ± 4.82 jk	15.3 ± 1.79 l
PteGlu	0	ND	ND	ND	ND	ND	ND	ND
THF		ND	ND	ND	ND	ND	ND	ND
5mTHF		ND	ND	0.6 ± 0.06 b	ND	ND	3.1 ± 0.59 a	0.2 ± 0.20 b
5fTHF		2.0 ± 0.53 ab	1.4 ± 0.32 ab	1.2 ± 0.10 b	0.3 ± 0.02 c	1.8 ± 0.12 ab	2.1 ± 0.24 a	1.5 ± 0.14 ab
10fPteGlu		ND	ND	ND	ND	ND	ND	ND
Total folate (μg/100 g FW)		2.0 ± 0.53 b	1.4 ± 0.32 b	1.8 ± 0.08 b	0.3 ± 0.02 c	1.8 ± 0.12 b	5.2 ± 0.76 a	1.7 ± 0.06 b

PteGlu: pteroylmonoglutamic acid, THF: tetrahydrofolate, 5mTHF: 5-methyltetrahydrofolate, 5fTHF: 5-formyltetrahydrofolate, 10fPteGlu: 10-formylpteroylglutamic acid. Data are mean ± SD (n = 3). Data without a common letter in each test indicate significant (*p* < 0.05) differences between samples using the Tukey (HSD) test.

**Table 8:** TPC and FRAP of the studied BP.

Test	Day	S1	S2	S3	S4	Y1	Y2	Y3
TPC	0	10.1 ± 0.71 hij	13.3 ± 1.21 e	11.5 ± 0.41 efgh	19.1 ± 0.98 bc	11.8 ± 0.82 efgh	11.4 ± 0.29 efgh	16.9 ± 1.41 d
(mg GAE/g FW)	4	11.1 ± 0.67 fgh	13.0 ± 0.68 e	12.5 ± 0.44 efg	21.5 ± 1.27 a	12.66 ± 1.03 ef	12.8 ± 0.89 ef	19.9 ± 1.55 ab
	7	5.9 ± 0.34 l	9.2 ± 0.48 ijk	8.6 ± 0.65 jk	17.5 ± 1.24 cd	8.0 ± 0.31 k	10.7 ± 1.07 ghi	7.7 ± 0.08 kl
FRAP	0	184.0 ± 12.89 f	223.5 ± 22.44 de	180.3 ± 14.17 f	332.1 ± 18.25 b	207.1 ± 21.11 def	205.1 ± 8.55 def	295.4 ± 24.91 c
(μmol Fe^2+^/g FW)	4	191.7 ± 8.80 ef	211.4 ± 10.27 def	211.3 ± 10.81 def	399.1 ± 24.80 a	231.4 ± 14.01 d	233.7 ± 12.70 d	328.0 ± 24.66 b
	7	113.8 ± 6.43 h	132.4 ± 3.62 gh	146.7 ± 7.00 g	284.8 ± 17.79 c	137.9 ± 4.76 gh	180.6 ± 10.19 f	140.7 ± 8.71 gh

TPC: total phenolic content. FRAP: ferric reducing antioxidant power. GAE: gallic acid equivalents. Data are mean ± SD (n = 3). Data without a common letter in each test indicate significant (*p* < 0.05) differences between samples using the Tukey (HSD) test.

**Table 9:** Sensory descriptors for the studied BP at the end of storage by consensus of 12 panellists.

	S1	S2	S3	S4	Y1	Y2	Y3
Aroma	slightly fermented, stewed fruits, grape skin aroma	slightly fermented, grassy	woody, grape	slightly fermented, black plum, blackberry, sweet grassy note	slightly fermented, plum, mulberry	slightly fermented, preserved prunus, blackberry	slightly fermented, plum stewed fruits
Appearance	glossy, dark maroon surface, creased, partly green and pink flesh	small, dull crimson surface, blush pink flesh	small, glossy deep maroon smooth surface, translucent pink flesh	small, slightly glossy dark maroon surface, shrivelled, partly green and pink flesh	large, black glossy surface, creased, beetroot color flesh	small, dull brown surface, creased, beetroot color flesh	small, dull black surface, shrivelled, pink flesh
Texture	juicy, creamy, astringent	fibrous, firm, dry, astringent	smooth, soft, juicy, dissolving, drying	soft, slightly grainy, astringent	tough, firm, fibrous, dry	soft, juicy	chewy
Flavor	sour, apple, kiwi, plum, mulberry	sour, slightly sweet, blackberry, floral, woody	sweet, slight sour, mulberry, plum	sour, slight sweet, blackberry, plum, stewed fruits	sour, slightly fermented, bitter, woody, grape	stewed fruits, sweet spice, slight fermented, woody, dried prunus	sour, dried fruits, slight fermented, plum
Aftertaste	tart, astringent	astringent, drying	sweet, slightly drying	woody, drying	sour, stewed fruit, bitter, drying	salivating, bitter	tart, astringent, bitter

## Data Availability

Data is provided in the article and Supplementary Material.

## Supplementary Materials

The following supporting information can be downloaded at: https://www.mdpi.com/article/10.3390/molecules28041608/s1, Table S1: Quantification of detected compounds in Burdekin plum; Figure S1: Ion chromatograms of Burdekin plums extract (S1, S2, S3, S4, Y1, Y2, Y3) in positive and negative modes; Figure S2: Mass spectra of tentatively identified compounds; Figure S3: Experimental design; (TSS: total soluble solids, TA: titratable acidity). Reference [129] are cited in the supplementary materials.

## Sample Availability

Not available.
