# Supplementary material for: Quality Assessment of Burdekin Plum (Pleiogynium timoriense) during Ambient Storage
Source: Molecules. 2023 Feb 7;28(4):1608. doi: 10.3390/molecules28041608 (PMC9958931; doi:10.3390/molecules28041608)
Supplement: Supplementary file 1 [file molecules-28-01608-s001.zip › molecules-2107321-supplementary.pdf]

Quality assessment of Burdekin plum (*Pleiogynium timoriense*) during ambient storage

Supplementary materials

Table S1. Quantification of detected compounds in Burdekin plum

Compound	Da	S1	S2	S3	S4	Y1	Y2	Y3
Delphinidin 3-galactoside	0	17.8 ± 0.21 h	28.0 ± 0.24 h	40.5 ± 0.4 gh	15.3 ± 0.1 h	236.0 ± 2.52 e	1188.1 ± 34.53 b	170.9 ± 3.27 f
	4	19.9 ± 1.24 h	29.9 ± 0.11 gh	81.2 ± 1.41 g	19.7 ± 0.31 h	266.4 ± 10.19 e	1213.8 ± 29.06 b	238.2 ± 1.38 e
	7	19.1 ± 0.39 h	27.6 ± 0.45 h	46.9 ± 1.06 gh	20.0 ± 0.3 h	150.7 ± 5.28 f	1125.7 ± 21.58 c	154.2 ± 0.93 f
Delphinidin 3-glucoside	0	0.8 ± 0.01 e	0.8 ± 0.01 e	1.0 ± 0.03 e	0.7 ± 0.01 e	3.5 ± 0.23 de	60.1 ± 1.55 b	5.5 ± 0.26 cd
	4	0.9 ± 0.04 e	0.9 ± 0.01 e	1.5 ± 0.14 e	0.8 ± 0.01 e	3.6 ± 0.20 de	68.8 ± 2.6 a	7.2 ± 0.19 c
	7	0.9 ± 0.04 e	1.0 ± 0.02 e	1.3 ± 0.04 e	0.9 ± 0.00 e	3.4 ± 0.13 de	63.2 ± 1.35 b	3.5 ± 0.12 de
Cyanidin 3-galactoside	0	597.9 ± 8.55 jk	212.8 ± 1.75 n	595.3 ± 30.1 jk	483.5 ± 12.23 l	1053.8 ± 24.18 c	789.9 ± 14.63 efg	838.5 ± 23.33 def
	4	646.8 ± 7.3 ij	534.9 ± 37.31 kl	845.1 ± 14.65 de	692.0 ± 28.08 hi	1366.0 ± 25.97 a	885.2 ± 43.94 d	1188.0 ± 28.78 b
	7	561.1 ± 13.96 k	355.2 ± 7.87 m	903.5 ± 16.17 d	767.8 ± 15.11 fg	987.3 ± 17.15 c	887.8 ± 8.75 d	1240.2 ± 26.63 b
Cyanidin 3-glucoside	0	5.1 ± 0.08 jkl	06 ± 0.07 m	2.2 ± 0.2 lm	3.7 ± 0.19 klm	22.0 ± 2.18 d	34.6 ± 1.81 b	10.6 ± 0.78 ghi
	4	14.0 ± 1.13 efg	15.7 ± 0.06 ef	7.8 ± 1.01 ij	10.3 ± 0.78 ghi	31.6 ± 0.97 b	49.1 ± 0.78 a	22.3 ± 1.81 d
	7	12.3 ± 1.06 fgh	7.5 ± 0.37 ijk	9.9 ± 0.54 hi	15.2 ± 1.1 ef	26.7 ± 2.10 c	45.4 ± 0.49 a	17.8 ± 2.60 e
Cyanidin 3-arabinoside	0	1.2 ± 0.01 no	0.8 ± 0.01 o	2.9 ± 0.08 kl	1.9 ± 0.07 m	3.5 ± 0.01 jk	2.9 ± 0.04 l	3.0 ± 0.15 kl
	4	1.6 ± 0.01 mn	3.7 ± 0.12 ij	5.0 ± 0.08 ef	4.0 ± 0.33 hi	5.1 ± 0.04 ef	5.0 ± 0.2 ef	5.0 ± 0.13 ef
	7	2.8 ± 0.04 l	4.3 ± 0.01 gh	15.4 ± 0.2 a	6.1 ± 0.05 c	5.4 ± 0.15 de	6.0 ± 0.14 c	5.8 ± 0.16 cd
Peonidin 3-hexosides	0	1.8 ± 0.02 g	1.5 ± 0.01 j	1.4 ± 0.01 l	1.5 ± 0.01 jk	1.8 ± 0.01 g	1.3 ± 0 m	1.5 ± 0.01 j
	4	2.5 ± 0.03 bc	2.5 ± 0.03 b	1.7 ± 0.01 hi	2.2 ± 0.06 e	2.3 ± 0.01 d	1.4 ± 0.01 kl	1.8 ± 0.01 g
	7	2.5 ± 0.01 c	2.6 ± 0.05 a	2.3 ± 0.05 d	2.5 ± 0.03 bc	2.1 ± 0.01 f	1.6 ± 0.01 ij	2.2 ± 0.01 e
Total anthocyanin	0	624.5 ± 8.41 lm	244.5 ± 1.95 p	643.3 ± 30.56 klm	506.6 ± 12.48 n	1320.5 ± 21.46 g	2076.9 ± 39 c	1030.1 ± 21.27 i
	4	685.7 ± 7.61 klm	587.6 ± 37.56 mn	942.2 ± 16.29 i	729.0 ± 29.37 jk	1675.0 ± 15.75 e	2223.3 ± 67.33 b	1462.5 ± 29.62 f
	7	598.6 ± 15.01 mn	398.2 ± 8.17 o	979.3 ± 16.32 i	812.5 ± 15.8 j	1175.6 ± 23.57 h	2129.6 ± 25.98 bc	1423.6 ± 25.2 fg
Citric acid	0	15901.0 ± 250.08 ghi	16532.8 ± 112.25 fg	14013.6 ± 74.97 jkl	15335.1 ± 137.74 i	15732.6 ± 103.84 hi	13155.1 ± 191.38 m	17611.8 ± 186.23 de
	4	17067.6 ± 150.02 ef	17863.0 ± 65.63 d	15357.8 ± 99.42 i	18516.2 ± 176.42 c	17528.1 ± 222.6 de	14040.1 ± 223.64 jkl	18729.0 ± 145.02 bc
	7	16364.9 ± 90.74 gh	18937.7 ± 63.23 bc	15687.0 ± 282.04 i	19322.7 ± 420.96 b	17442.2 ± 521.43 de	13850.7 ± 155.38 kl	23341.0 ± 155.69 a
Malic acid	0	1824.0 ± 66.61 b	2338.9 ± 58.56 a	1615.5 ± 35.71 c	1634.3 ± 19.39 c	567.3 ± 23.65 m	1256.4 ± 39.56 efg	968.8 ± 31.96 ij
	4	1985.1 ± 33.8 b	2170.9 ± 82.13 a	1397.6 ± 97.55 def	1538.7 ± 38.75 cd	492.6 ± 7.99 m	1131.1 ± 35.6 ghi	848.1 ± 44.47 jk
	7	1367.0 ± 31.02 def	1911.6 ± 41.72 b	1238.1 ± 18.5 efg	1158.0 ± 74.89 gh	596.8 ± 12.29 lm	845.6 ± 9.93 jk	765.5 ± 12.93kl
Quinic acid	0	987.9 ± 32.93 n	1926.4 ± 36.89 fg	2344.4 ± 105.98 d	2518.5 ± 84.16 cd	1632.7 ± 18.36 ij	1268.1 ± 41.57 lm	1460.9 ± 40.73 jkl
	4	1104.1 ± 7.41 mn	1843.6 ± 37.57 gh	2095.1 ± 75.7 ef	2425.2 ± 76.48 cd	1340.6 ± 17.56 kl	1286.5 ± 28.97 lm	1278.6 ± 42.41 lm
	7	1431.0 ± 51.6 jkl	2129.2 ± 44.6 e	3361.8 ± 117.13 a	2878.67 ± 116.83 b	1678.5 ± 47.01 hi	1469.6 ± 14.95 jkl	2051.5 ± 44.38 ef
Gallic acid	0	12.6 ± 0.2 ij	2.3 ± 0.17 o	9.4 ± 0.09 jkl	50.4 ± 0.27 f	5.2 ± 0.13l mno	4.5 ± 0.18 no	9.4 ± 0.38 jkl
	4	15.2 ± 0.3 hi	2.8 ± 0.04 o	9.2 ± 0.17 jklm	38.6 ± 0.42 g	10.6 ± 0.26 jk	7.7 ± 0.12 klmn	7.3 ± 0.17 klmn
	7	59.9 ± 0.51 e	7.6 ± 0.16 klmn	11.9 ± 0.29 ijk	131.9 ± 4.61 c	63.2 ± 0.7 e	210.1 ± 4.11 a	144.8 ± 1.13 b
Quercetin 3-glucoside	0	1.9 ± 0.08 efgh	6.8 ± 0.13 b	1.0 ± 0.11 kl	1.1 ± 0.07 jkl	1.9 ± 0.1 ef	1.6 ± 0.08 fghi	2.7 ± 0.09 c
	4	2.8 ± 0.25 c	6.5 ± 0.28 b	1.9 ± 0.18 efgh	2.0 ± 0.09 efg	2.2 ± 0.17 d e	1.4 ± 0.14 ij	2.6 ± 0.08 c
	7	1.6 ± 0.02 fghi	8.0 ± 0.1 a	1.5 ± 0.1 ij	1.3 ± 0.06 ijk	1.5 ± 0.09 hij	1.5 ± 0.02 fghi	2.5 ± 0.04 cd
Quercetin	0	1.8 ± 0.00 gh	0.5 ± 0.01 o	1.7 ± 0.01 hi	1.6 ± 0.03 i	0.7 ± 0.01 n	0.7 ± 0.01n	1.4 ± 0.02 j
	4	3.8 ± 0.04 a	0.8 ± 0.03 mn	2.2 ± 0.03 d	2.2 ± 0.07 d	1.0 ± 0.02 l	0.8 ± 0.03m	2.0 ± 0.03 e

	7	3.0 ± 0.05 b	1.1 ± 0.02 k	2.7 ± 0.1 c	2.6 ± 0.04 c	1.4 ± 0.03 j	1.9 ± 0.03 f	2.2 ± 0.06 d
Ellagic acid	0	32.6 ± 1.84 mn	58.0 ± 4.62 mn	224.6 ± 2.09 k	381.2 ± 8.6 ij	1050.1 ± 15.77 c	771.6 ± 0.59 f	389.0 ± 6.42 i
	4	21.6 ± 2.23 n	127.1 ± 5.42 l	331.8 ± 6.69 j	526.66 ± 35.74 gh	1270.1 ± 56.29 a	965.2 ± 23.14 d	522.8 ± 24.03 gh
	7	36.8 ± 5.22 mn	82.2 ± 3.28 lm	360.3 ± 19.01 ij	487.5 ± 6.69 h	1061.3 ± 6.68 c	1119.9 ± 5.63 b	544.1 ± 13.92 g
Catechin	0	7.3 ± 0.31 hij	11.9 ± 0.49 d	2.2 ± 0.17 op	29.4 ± 1.27 b	2.8 ± 0.07 nop	4.6 ± 0.01 lmn	21.3 ± 0.09 c
	4	9.0 ± 0.2 fgh	11.2 ± 0.22 de	1.4 ± 0.16 p	39.5 ± 1.9 a	3.6 ± 0.07 mno	5.0 ± 0.2 klm	8.6 ± 0.3 ghi
	7	11.0 ± 0.25 de	9.9 ± 0.11 efg	4.3 ± 0.14 mn	40.6 ± 1.86 a	3.3 ± 0.11 mno	6.7 ± 0.13 ijk	10.9 ± 0.21 def
Epicatechin	0	8.1 ± 0.2 n	20.7 ± 0.29 ef	7.0 ± 0.06 n	15.2 ± 0.4 j	11.3 ± 0.3 l	15.4 ± 0.08 j	26.2 ± 0.4 c
	4	9.9 ± 0.16 m	20.6 ± 0.49 fg	6.8 ± 0.12 n	21.0 ± 1 ef	11.5 ± 0.06 l	17.3 ± 0.09 h	16.3 ± 0.11 hij
	7	13.7 ± 0.18 k	32.5 ± 0.99 a	16.9 ± 0.36 hi	22.0 ± 1.02 e	12.9 ± 0.35 k	19.3 ± 0.52 g	28.1 ± 0.51 b
1.3.6-trigalloylglucose	0	2.3 ± 0.3 lm	5.1 ± 0.13 i	2.2 ± 0.05 lm	5.1 ± 0.18 i	1.0 ± 0.14 n	13.6 ± 0.15 c	2.4 ± 0.11 lm
	4	2.6 ± 0.04 l	7.1 ± 0.04 fg	3.0 ± 0.05 kl	6.3 ± 0.36 gh	1.5 ± 0.1 mn	16.6 ± 0.63 b	3.7 ± 0.1 jk
	7	5.3 ± 0.04 hi	8.0 ± 0.24 f	4.6 ± 0.07 ij	9.3 ± 0.58 e	1.6 ± 0.43 mn	12.7 ± 0.91 c	2.8 ± 0.12 kl
Trigalloyl glucose isomer I&II&III	0	17.7 ± 0.82 g	10.2 ± 0.13 i	3.9 ± 0.31 l	29.0 ± 0.77 d	7.5 ± 0.28 jk	29.2 ± 1.83 d	8.1 ± 0.43 ij
	4	21.3 ± 0.60 f	15.5 ± 0.52 gh	5.5 ± 0.23 kl	32.6 ± 0.99 c	8.3 ± 1.07 ij	38.7 ± 0.74 a	9.7 ± 0.39 ij
	7	24.5 ± 0.55 e	13.8 ± 0.35 h	5.7 ± 0.26 kl	35.6 ± 0.5 b	5.5 ± 0.27 kl	25.2 ± 1.66 e	7.5 ± 0.19 jk
Trigalloyl glucose isomer IV&V	0	108.9 ± 1.51 e	19.8 ± 0.59 jk	58.3 ± 0.47 h	154.4 ± 2.61 c	23.1 ± 0.38 ij	12.8 ± 0.32 lmn	8.1 ± 0.2 n
	4	138.4 ± 0.82 d	28.0 ± 0.64 i	83.3 ± 0.35 f	178.5 ± 6.37 b	23.2 ± 0.54 ij	14.2 ± 0.18 klm	8.3 ± 0.14 n
	7	151.4 ± 2.18 c	24.1 ± 0.53 ij	73.9 ± 0.43 g	190.0 ± 3.82 a	17.0 ± 0.4 kl	10.9 ± 0.95 mn	9.4 ± 0.14 mn
Digalloyl glucose	0	201.1 ± 3.17 e	43.9 ± 2.46 g	196.3 ± 5.75 e	353.7 ± 10.51 b	59.4 ± 1.69 fg	46.2 ± 1.34 g	49.8 ± 2.77 fg
	4	279.6 ± 4.91 c	67.4 ± 2.19 f	260.0 ± 3.6 d	398.7 ± 19.51 a	61.0 ± 3.72 fg	56.9 ± 0.64 fg	53.1 ± 2.76 fg
	7	249.4 ± 5.1 d	53.9 ± 0.22 fg	213.5 ± 3.64 e	398.3 ± 5.45 a	45.0 ± 0.3 g	52.7 ± 0.97 fg	48.0 ± 0.83 g
Tetragalloyl glucose	0	18.3 ± 0.16 e	3.9 ± 0.11 hi	3.2 ± 0.04 i	22.6 ± 0.09 c	1.6 ± 0.03 j	5.3 ± 0.08 fg	1.6 ± 0.04 j
	4	20.7 ± 0.49 d	4.2 ± 0.07 ghi	4.5 ± 0.06 gh	27.1 ± 1.18 a	1.8 ± 0.03 j	6.1 ± 0.3 f	1.8 ± 0.05 j
	7	23.9 ± 0.74 b	4.6 ± 0.09 gh	4.1 ± 0.04 hi	26.2 ± 0.48 a	1.6 ± 0.03 j	4.2 ± 0.18 hi	1.7 ± 0.05 j
5-galloylquinic acid	0	116.6 ± 0.2 fgh	6.9 ± 0.08 k	172.0 ± 0.34 e	99.1 ± 0.63 i	74.7 ± 1.69 j	102.4 ± 0.99 i	179.7 ± 0.97 d
	4	110.4 ± 1.28 h	7.0 ± 0.17 k	183.7 ± 1.87 d	119.7 ± 2.16 f	79.2 ± 1.27 j	115.9 ± 1.45 fgh	194.1 ± 2.06 c
	7	117.5 ± 1.26 fg	8.5 ± 0.02 k	213.0 ± 1.12 a	119.3 ± 4.65 fg	75.2 ± 0.53 j	113.1 ± 5.27 gh	203.9 ± 3.14 b
4-galloylquinic acid	0	5.0 ± 0.04 hi	3.3 ± 0.06 j	0.5 ± 0.01 k	42.9 ± 0.26 c	6.5 ± 0.12 fgh	5.2 ± 0.04 ghi	20.0 ± 0.41 e
	4	4.1 ± 0.03 ij	3.1 ± 0.06 j	0.5 ± 0.01 k	45.3 ± 1.19 b	6.3 ± 0.11 fgh	6.1 ± 0.15 fgh	20.1 ± 0.2 e
	7	5.0 ± 0.02 hi	3.7 ± 0.01 ij	0.8 ± 0.02 k	52.4 ± 1.78 a	6.8 ± 0.12 f	6.6 ± 0.18 fg	21.9 ± 0.56 d
Quercetin 3-glucoside isomer	0	10.9 ± 0.07 g	16.4 ± 0.1 c	11.0 ± 0.32 g	3.84 ± 0.02 k	11.2 ± 0.13 g	9.1 ± 0.08 h	9.3 ± 0.15 h
	4	13.5 ± 0.12 ef	17.4 ± 0.46 bc	14.3 ± 0.22 de	5.6 ± 0.41 j	14.8 ± 0.3 d	10.6 ± 0.61 g	15.1 ± 0.24 d
	7	11.2 ± 0.01 g	22.6 ± 0.2 a	17.9 ± 0.98 b	6.8 ± 0.11 i	13.1 ± 0.2 f	13.0 ± 0.35 f	17.2 ± 0.31 bc
Quercetin glucuronide	0	9.6 ± 0.13 g	33.9 ± 0.31 b	13.8 ± 0.12 d	3.9 ± 0.08 j	4.2 ± 0.1 j	1.6 ± 0.02 l	8.1 ± 0.09 h
	4	10.1 ± 0.23 fg	30.0 ± 0.68 c	11.8 ± 0.31 e	5.2 ± 0.15 i	4.6 ± 0.11 ij	2.0 ± 0.09 kl	9.8 ± 0.15 fg
	7	7.4 ± 0.11 h	36.0 ± 0.59 a	13.2 ± 0.67 d	4.3 ± 0.06 j	2.8 ± 0.04 k	2.1 ± 0.1 kl	10.5 ± 0.36 f
	0	20.2 ± 0.3 fgh	15.6 ± 0.07 jk	21.8 ± 0.16 efg	71.4 ± 0.93 a	18.6 ± 0.4 hi	14.5 ± 0.28 k	23.7 ± 0.21 de
	4	24.5 ± 0.54 de	20.3 ± 0.43 fgh	22.9 ± 0.49 ef	67.4 ± 3.14 b	21.9 ± 0.45 efg	20.6 ± 0.27 fgh	20.0 ± 0.43 gh

Epicatechin	7	26.4 ± 0.26 d	18.3 ± 0.16 hij	35.6 ± 0.72 c	69.2 ± 1.99 ab	16.8 ± 0.15 ijk	15.4 ± 0.55 k	19.9 ± 0.32 gh
Luteolin glucuronide	0	6.7 ± 0.05 cd	17.1 ± 0.15 b	1.6 ± 0.04 lm	2.3 ± 0.05 jk	3.9 ± 0.07 fg	0.3 ± 0.01 n	3.5 ± 0.04 gh
	4	7.1 ± 0.17 c	17.5 ± 0.72 ab	1.5 ± 0.07 m	3.0 ± 0.21 hi	4.3 ± 0.07 f	0.6 ± 0.01 n	4.2 ± 0.05 f
	7	6.4 ± 0.17 d	18.0 ± 0.21 a	1.2 ± 0.05 m	2.1 ± 0.07 kl	2.8 ± 0.11 ij	0.5 ± 0.03 n	5.0 ± 0.11 e

Results were expressed in mg/kg FW. Some compounds were semiquantified using related standards (Cyanidin 3-arabinoside used cyanidin 3-glucoside; peonidin 3-hexoside used delphinidin 3-glucoside; Digalloyl glucose, galloyl quinic acid, epicatechin gallate used gallic acid; tetragalloyl glucose used trigalloyl glucose; (epi)catechin gallate used epicatechin; quercetin glucuronide used quercetin 3-glucoside; luteolin glucuronide used luteolin as standard), therefore the quantification result of these compounds was only an estimation because of the differences in the response of these compounds compared to the standards used (Gómez-Caravaca et al., 2016) [129]. Data without a common letter in each test indicate significant ($p < 0.05$) differences between samples

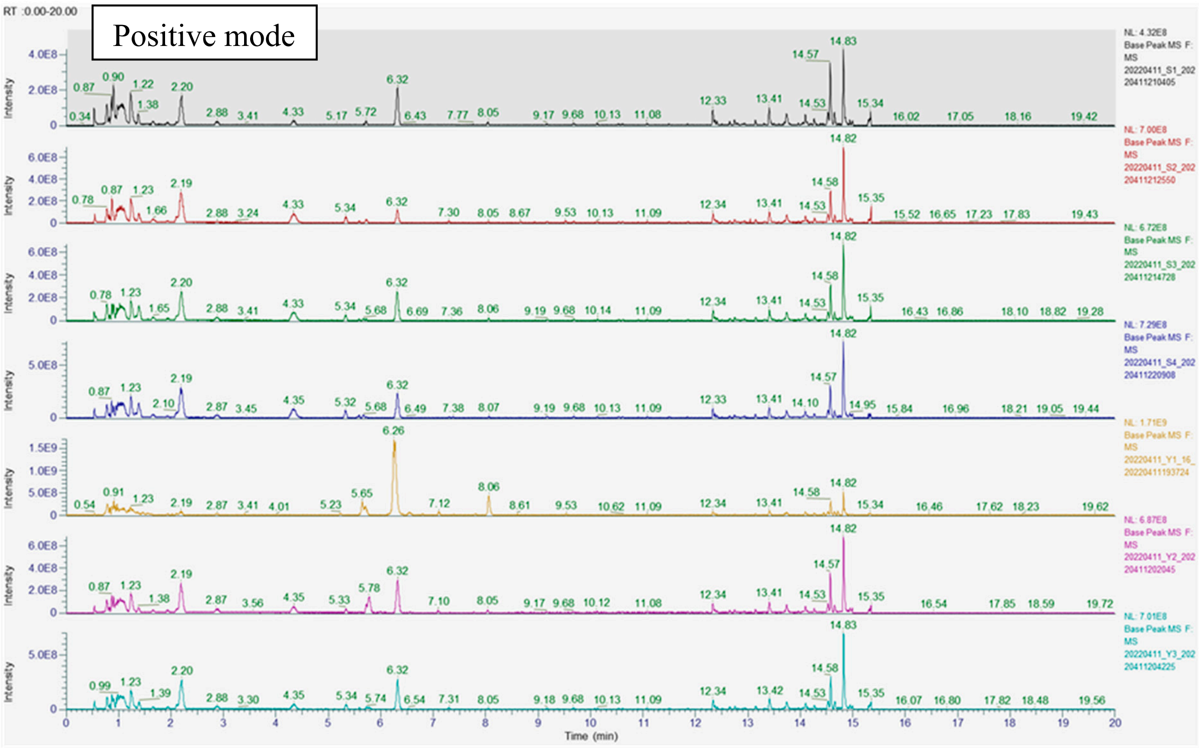
RT :0.00-20.00
Positive mode
Intensity
Time (min)

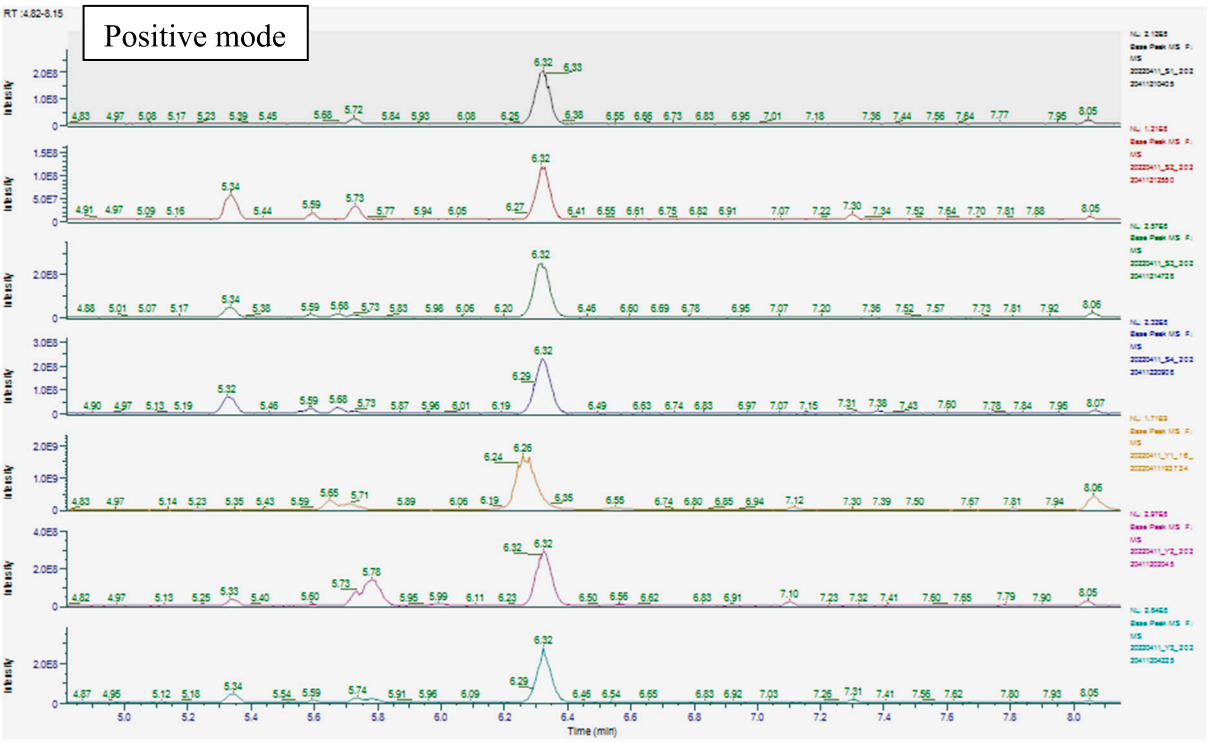
RT :4.82-8.15
Positive mode
Time (min)

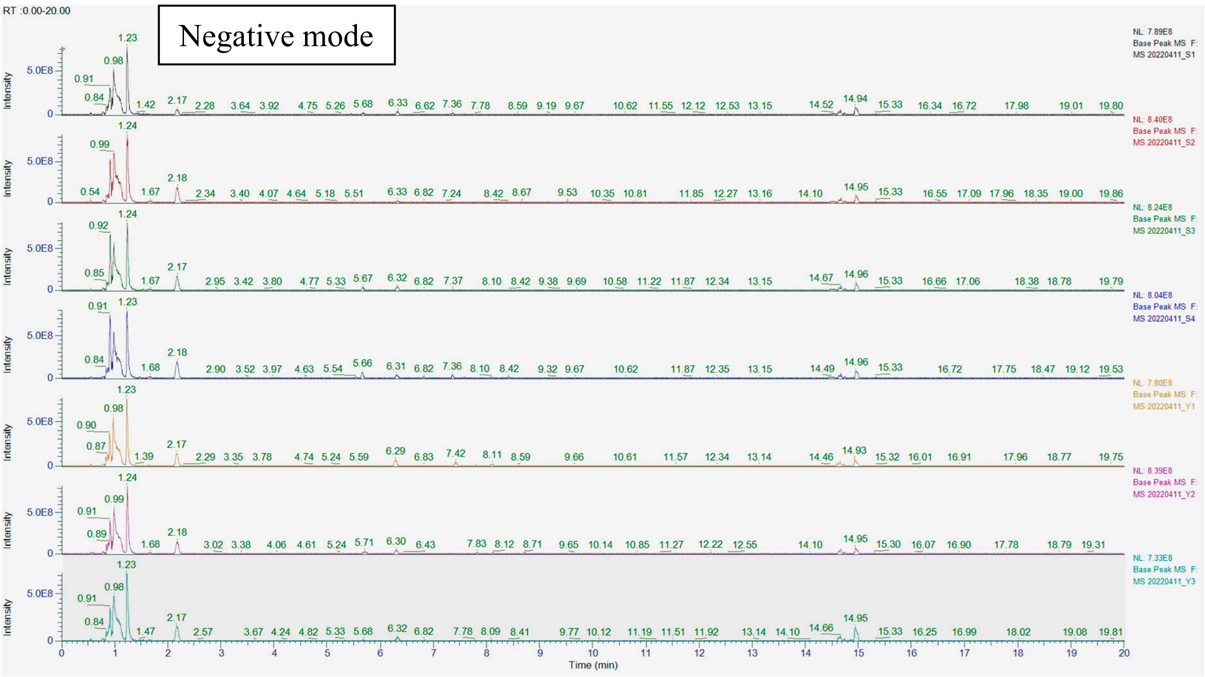

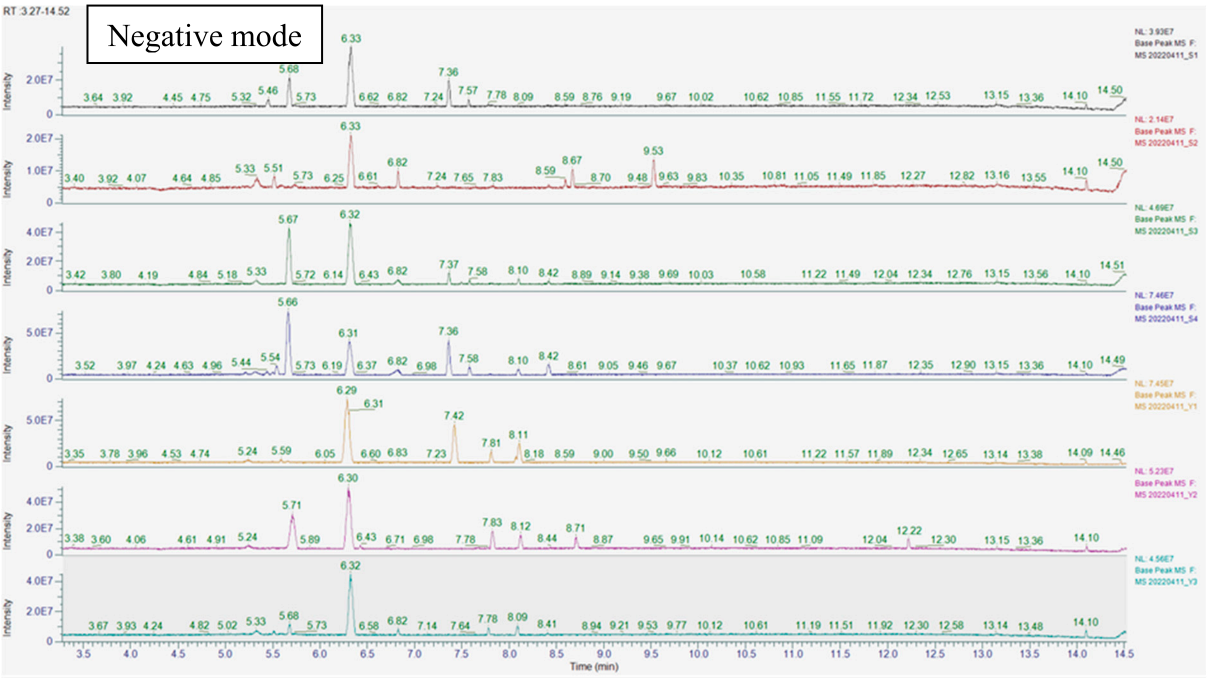

Figure S1. Ion chromatograms of Burdekin plums extract (S1,S2,S3,S4,Y1,Y2,Y3) in positive and negative modes

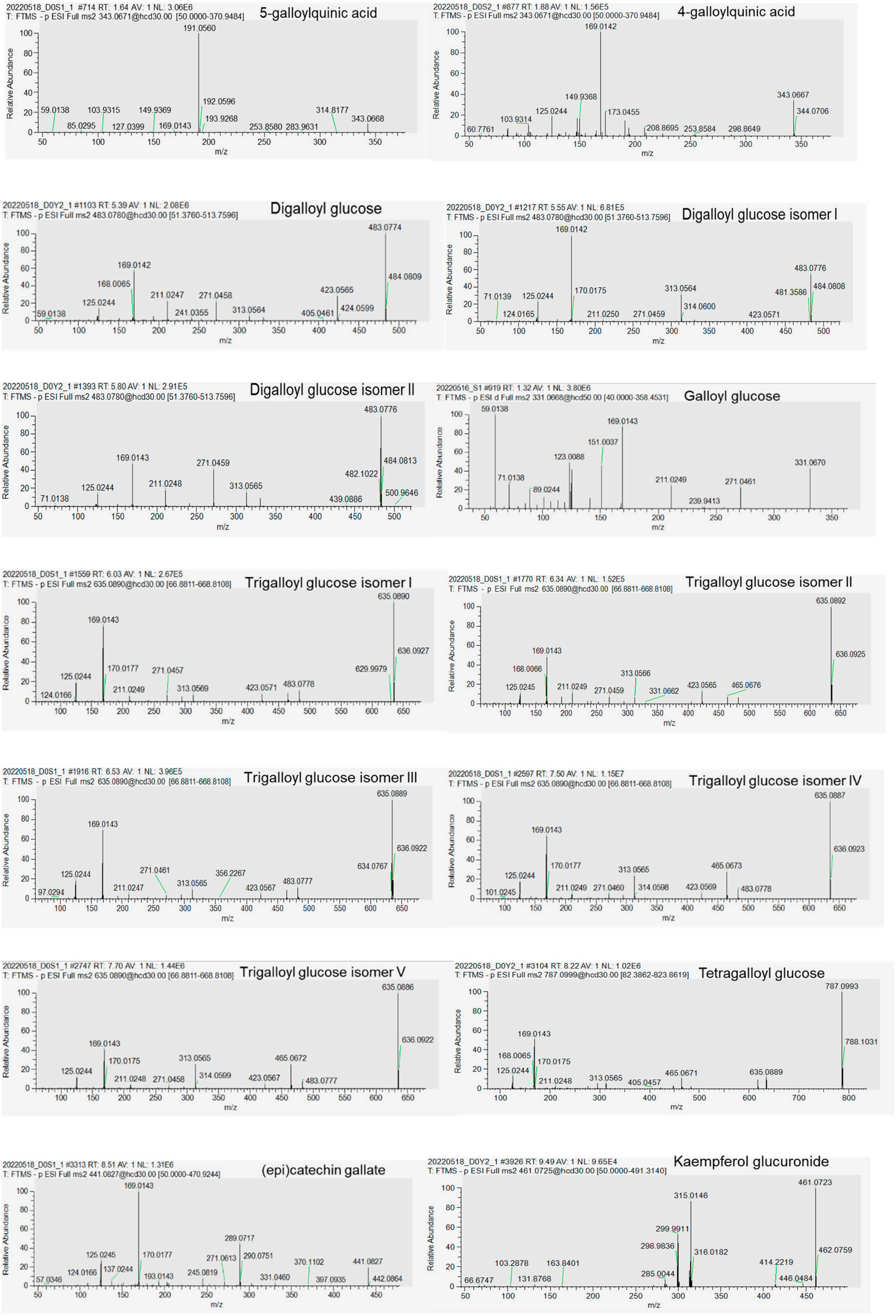
5-galloylquinic acid
4-galloylquinic acid
Digalloyl glucose
Digalloyl glucose isomer I
Digalloyl glucose isomer II
Galloyl glucose
Trigalloyl glucose isomer I
Trigalloyl glucose isomer II
Trigalloyl glucose isomer III
Trigalloyl glucose isomer IV
Trigalloyl glucose isomer V
Tetragalloyl glucose
(epi)catechin gallate
Kaempferol glucuronide

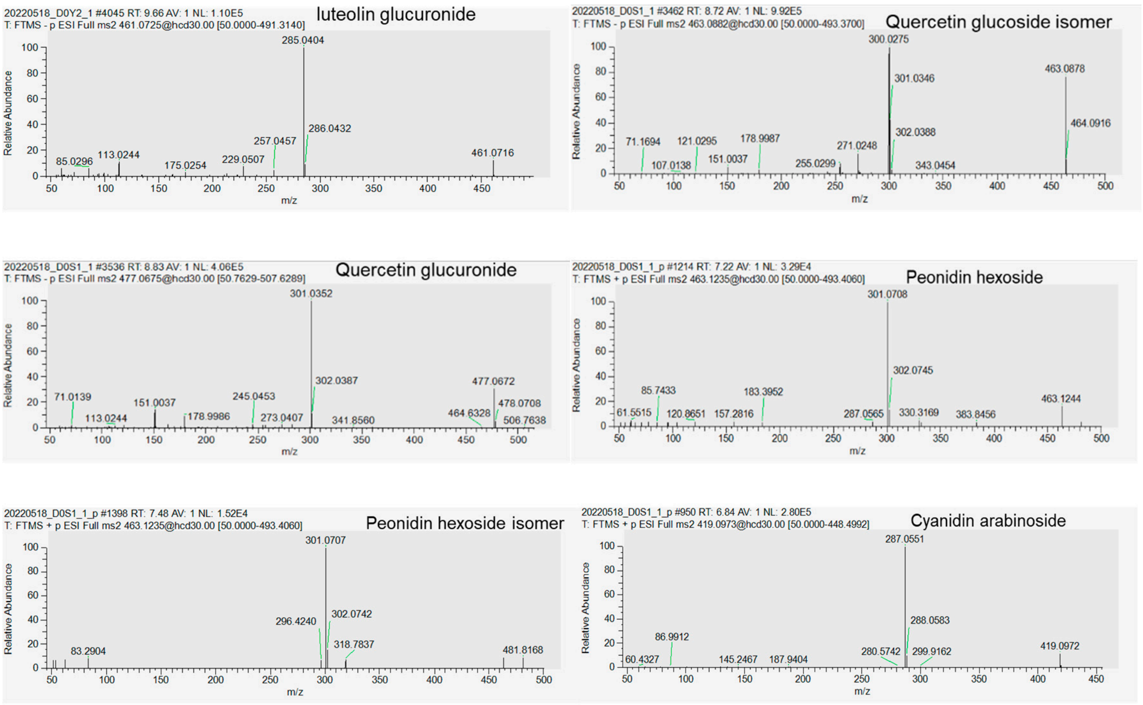

Figure S2. Mass spectra of tentatively identified compounds in BP

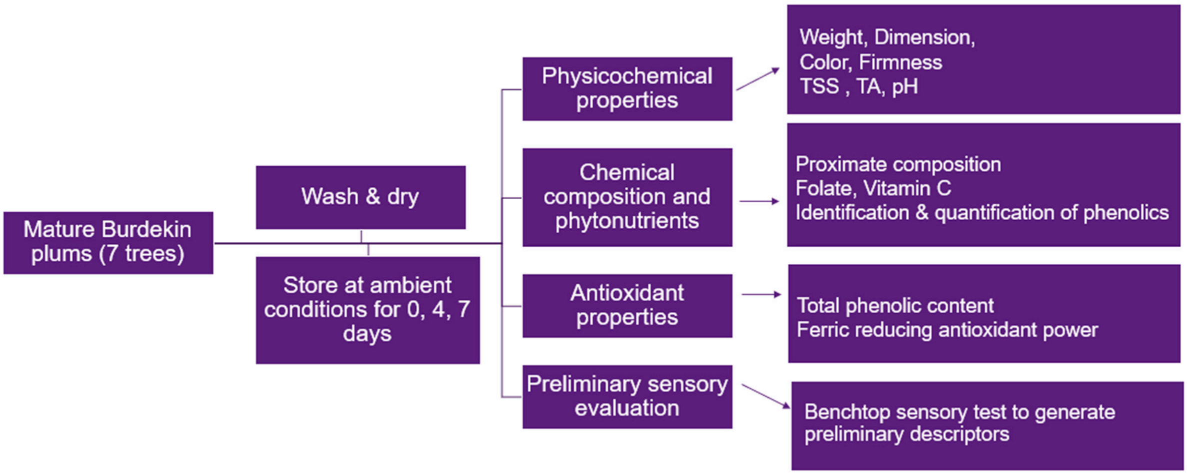

Figure S3. Experimental design; (TSS: total soluble solids, TA: titratable acidity)
